# The identification of alternative oxidase in intermediate host snails of *Schistosoma* and its potential role in protecting *Oncomelania hupensis* against niclosamide-induced stress

**DOI:** 10.1186/s13071-022-05227-5

**Published:** 2022-03-21

**Authors:** Ni Jiang, Shi-Zhu Li, Yang-Wen-Qing Zhang, Mohamed R. Habib, Tao Xiong, Sha Xu, Huifen Dong, Qin-Ping Zhao

**Affiliations:** 1 Department of Parasitology, School of Basic Medical Sciences, Wuhan University, Wuhan, Hubei China; 2 National Institute of Parasitic Diseases, Chinese Center for Disease Control and Prevention, National Center for Tropical Diseases Research, WHO Collaborating Center for Tropical Diseases, Key Laboratory of Parasite and Vector Biology, Ministry of Health, Shanghai, China; 3 Joint Inspection Center of Precision Medicine, The People’s Hospital of Guangxi Zhuang Autonomous Region, Nanning, Guangxi China; 4 Medical Malacology Department, Theodor Bilharz Research Institute, Giza, Egypt; 5 Department of Microbiology, School of Medical Sciences, Hunan University of Chinese Medicine, Changsha, Hunan China

**Keywords:** Alternative oxidase, Niclosamide, *Oncomelania hupensis*, Intermediate host, *Schistosoma*, Mitochondrial respiratory chain

## Abstract

**Background:**

Snail intermediate hosts are mandatory for the transmission of schistosomiasis, which has to date infected more than 200 million people worldwide. Our previous studies showed that niclosamide treatment caused the inhibition of aerobic respiration and oxidative phosphorylation, and the disruption of energy supply, in one of the intermediate hosts of schistosomiasis, *Oncomelania hupensis*, which eventually led to the death of the snails. Meanwhile, the terminal oxidase in the mitochondrial respiratory chain, alternative oxidase (AOX), was significantly up-regulated, which was thought to counterbalance the oxidative stress and maintain metabolic homeostasis in the snails. The aims of the present study are to identify the AOXs in several species of snails and investigate the potential activation of *O. hupensis AOX* (*OhAOX*) under niclosamide-induced stress, leading to enhanced survival of the snail when exposed to this molluscicide.

**Methods:**

The complete complementary DNA was amplified from the AOXs of *O. hupensis* and three species of *Biomphalaria*; the sequence characteristics were analysed and the phylogenetics investigated. The dynamic expression and localisation of the *AOX* gene and protein in *O. hupensis* under niclosamide-induced stress were examined. In addition, the expression pattern of genes in the mitochondrial respiratory complex was determined and the production of reactive oxygen species (ROS) calculated. Finally, the molluscicidal effect of niclosamide was compared between snails with and without inhibition of AOX activity.

**Results:**

AOXs containing the invertebrate AOX-specific motif NP-[YF]-XPG-[KQE] were identified from four species of snail, which phylogenetically clustered together into Gastropoda AOXs and further into Mollusca AOXs. After niclosamide treatment, the levels of *OhAOX* messenger RNA (mRNA) and OhAOX protein in the whole snail were 14.8 and 2.6 times those in untreated snails, respectively, but varied widely among tissues. Meanwhile, the level of cytochrome C reductase mRNA showed a significant decrease in the whole snail, and ROS production showed a significant decrease in the liver plus gonad (liver-gonad) of the snails. At 24 h post-treatment, the mortality of snails treated with 0.06–0.1 mg/L niclosamide and AOX inhibitor was 56.31–76.12% higher than that of snails treated with 0.1 mg/L niclosamide alone.

**Conclusions:**

AOX was found in all the snail intermediate hosts of *Schistosoma* examined here. AOX was significantly activated in *O. hupensis* under niclosamide-induced stress, which led to a reduction in oxidative stress in the snail. The inhibition of AOX activity in snails can dramatically enhance the molluscicidal effect of niclosamide. A potential target for the development of an environmentally safe snail control method, which acts by inhibiting the activity of AOX, was identified in this study.

**Graphical abstract:**

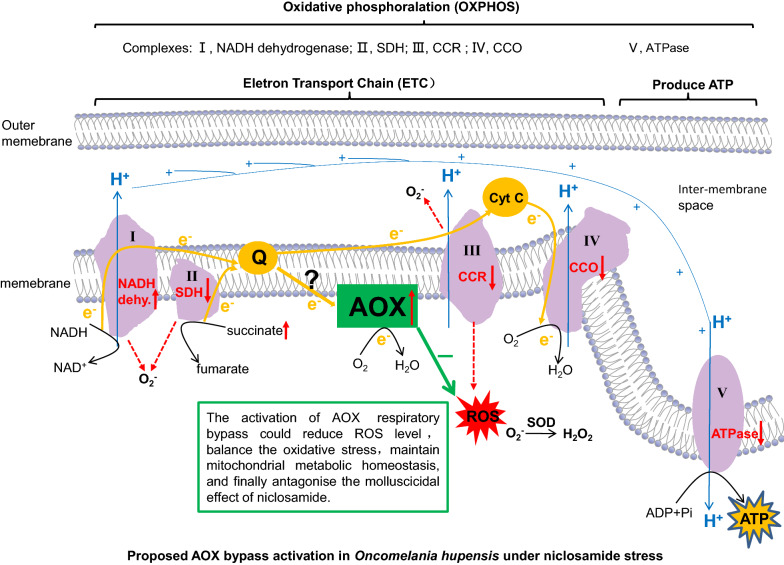

**Supplementary Information:**

The online version contains supplementary material available at 10.1186/s13071-022-05227-5.

## Background

Schistosomiasis is a widespread zoonotic disease in tropical and subtropical regions, and the second most prevalent parasitic disease in the world [1]. Seven species of *Schistosoma* can parasitise humans, and all of them are transmitted through contact between the skin and their cercaria, which are released from the specific intermediate snail host [2]. Since snails are obligatory intermediate hosts in the life cycle of *Schistosoma*, snail control is one of the most important measures used to block schistosomiasis transmission, including in areas where schistosomiasis elimination is the aim, such as in areas endemic for* Schistosomiasis japonicum* in China. Although the infection rates in humans and livestock in China remain low, high-risk factors for schistosomiasis transmission still exist, such as an increasing number of imported cases of schistosomiasis [3], the spread of *Biomphalaria straminea* (the intermediate host of *Schistosoma mansoni* in South America) in south China [4], wild animals as sources of infection [5], and the large geographical distribution of intermediate host snails of the genus *Oncomelania* [6]. *Oncomelania hupensis* is the only intermediate host of *S. japonicum*. In the past 20 years, the distribution of *O. hupensis* in China has not significantly reduced, and the population has remained at approximately 3.52–3.86 billion m^2^ [7]. In addition, the distribution of the snail in some areas endemic for schistosomiasis has shown an increasing trend as a consequence of ecological and environmental changes [6, 8].

Among the various approaches for snail control, chemical molluscicides are widely used due to their high potential coverage, the simplicity of their application, and their fast action [9]. Niclosamide is the only chemical molluscicide recommended by the World Health Organization, and has been used in areas of China endemic for schistosomiasis for nearly 30 years [10]. Some other chemicals and plant extracts, such as niclosamidate and luo-wei, were reported to have considerable molluscicidal effects with limited toxicity to aquatic animals [11, 12]. However, these compounds are not as effective as niclosamide, and logistic difficulties may arise during their application in the field [11, 12]. Niclosamide, therefore, has remained the main molluscicide for snail control. Although snails of the genus *Oncomelania* have not shown apparent resistance to niclosamide, there is a potential risk of resistance due to the long-term and extensive use of this single molluscicide [13]. In addition, niclosamide is highly toxic to fish and other aquatic animals. Thus, a deeper understanding of the response of *Oncomelania* to niclosamide is required to enable the development of an environmentally safe method for the highly efficient and specific control of these snails.

A previous study [14] showed that niclosamide can damage the liver and gonad (liver-gonad), ganglion, and muscle tissues of *O. hupensis*, with systems such as those of the mitochondrion, endoplasmic reticulum, and nucleus being the most affected. The mitochondrion is an organelle central to the production of chemical energy; it is involved in carbohydrate, amino acid and fatty acid metabolism, redox homeostasis, and cell signal transduction pathways, and is responsible for more than 90% of the adenosine triphosphate (ATP) produced in the cells of animals [15, 16]. Our previous studies on the enzymatic histochemistry and metabolic changes of *O. hupensis* in response to niclosamide revealed that the activities of cytochrome c oxidase (CCO), succinic dehydrogenase (SDH), and glucose-6-phosphatase in the respiratory chain of mitochondrial oxidative phosphorylation decreased after niclosamide treatment. In contrast, lactate dehydrogenase activity in the anaerobic glycolysis pathway increased [14, 17]. Niclosamide interfered with the metabolism of snails, damaged the structure of tissues, and led to the death of *O. hupensis* [14, 17]. In addition, the transcriptomic analysis of genes involved in mitochondrial oxidative phosphorylation revealed that nicotinamide adenine dinucleotide dehydrogenase subunit 3 was up-regulated and Na/K transporter ATPase subunit α was down-regulated after niclosamide treatment [18]. This indicated that niclosamide might induce oxidative stress by inhibiting the cytochrome c (Cyt c) pathway, i.e. aerobic respiration [14, 18]. Meanwhile, alternative oxidase (AOX), which is the terminal oxidase of a respiratory bypass of the mitochondrion [19], was significantly up-regulated [14, 18]. AOX is found in higher plants, algae, fungi, and protists, but not in vertebrates [19, 20]. It is involved in the generation of heat [19, 21], the optimisation of photosynthesis [22], the prevention of excessive reactive oxygen species (ROS) formation [23, 24], resistance to stress [24–26], and the maintenance of cellular energy homeostasis [26]. These functions of AOX were mostly found in plants. It is believed that the AOX bypass can accept electrons from ubiquinone, thereby reducing the membrane potential and preventing the over-reduction of ubiquinone in the respiratory chain from reducing ROS production [23, 24]. This occurs not only in plants but also in fungi and protozoa. Production of ROS increased in *Aspergillus fumigatus* after AOX was silenced [27]. H_2_O_2_ production in *Acanthamoeba castellanii* reduced significantly when the mitochondrial AOX respiratory pathway was activated, and this reduction was reversed when AOX was inhibited [28]. AOX in the molluscs *Crassostrea virginica* and *Diplodon chilensis* also played a role in resistance to stress [29, 30]. These findings led us to deduce that the activated AOX bypass could sustain metabolic homeostasis in *O. hupensis* under niclosamide-induced stress while the Cyt c pathway was inhibited [14, 17, 18], reduce cell damage caused by ROS accumulation in the mitochondrion [14, 17, 18], and weaken the molluscicidal effect of niclosamide.

First, this study proved the existence of AOX in the intermediate snail hosts of different *Schistosoma* species, including *O. hupensis* and three species of *Biomphalaria.* The biological characteristics of the AOXs of these four snail species and the phylogenetic evolution of the mollusc* AOX*s based on their sequences were then investigated. After niclosamide treatment, the dynamic expression of *O. hupensis* AOX (*OhAOX*) and tissue localisation of OhAOX were checked by measuring mRNA and protein levels. Also, the dynamic expression of genes involved in oxidative phosphorylation was investigated, and the ROS level was evaluated. Finally, snail mortality was determined in a molluscicidal assay with or without OhAOX inhibition to verify the resistance of OhAOX to niclosamide-induced stress and evaluate the possibility that OhAOX can be used as a molluscicidal enhancer target.

## Methods

### Snail collection

*Oncomelania hupensis* snails were collected on several occasions from habitats with different environmental conditions in a location (30.0011°N, 111.9716°E) in Gong’an county, Hubei Province, China, under the permission and assistance of the local snail control organisation, the Institute of Schistosomiasis Control of Gong’an County. No infected snail has been found in the area for years, and no molluscicide has been used there. The snails were brought back to the laboratory and rinsed with dechlorinated tap water. Snails with higher crawling activity that ranged in shell height from 6 to 8 mm were selected to ensure uniformity. These were maintained on wet rough filter paper with antibiotics in Petri dishes covered with plastic mesh, and then placed in an incubator at 25 °C with fresh humidified air. The snails were fed and the filter papers changed every 3 days for a month to allow them to adapt to the laboratory conditions.

*Biomphalaria glabrata* snails that have been kept and bred for a period of 10 years in Dr Yousheng Liang’s laboratory at Jiangsu Provincial Institute of Schistosomiasis Control and Prevention, were kindly provided for this study. *Biomphalaria alexandrina* snails were collected from a location (30.0802°N, 31.0962°E) in Teraat Al Mansoureya, Abu Rawash, Giza, Egypt, with the assistance of the Department of Medical Malacology, Theodor Bilharz Research Institute. *Biomphalaria straminea* snails were collected from a location (22.5794°N, 113.9504°E) in Shenzhen city, Guangdong Province, China, under the permission and assistance of the Shenzhen Center for Disease Control and Prevention. The snails were brought back to the laboratory, rinsed with dechlorinated tap water, and prepared for the experiments.

### Amplification, identification, and phylogenetic analysis of snail *AOX*s

#### BLAST analysis and selection of* AOX* fragments

Based on our previous work [18], a BLAST database was constructed using local sequence alignment of the *O. hupensis* transcriptomic unigene (NCBI SRA accession no. SRP041729) using ncbi-blast-2.8.1+ (ftp://ftp.ncbi.nlm.nih.gov/blast/executables/blast+). Each *AOX*-related unigene filtered out in a Swiss-Prot database analysis with an E-value of < 10^–9^ was checked, and 23 meaningful fragments were then obtained from the local BLAST analysis. Finally, the longest sequence was selected as the reference sequence for subsequent *OhAOX* complementary DNA (cDNA) amplification. The predicted *B. glabrata* AOX mRNA (GenBank accession no. XM_013230806) computed from a *B. glabrata* genomic sequence (NW_013418361.1) was chosen as the reference sequence for subsequent cDNA amplification of *B. glabrata AOX* (*BgAOX*), *B. alexandrina AOX* (*BaAOX*), and *B. straminea AOX* (*BsAOX*).

#### RNA extraction and* AOX* cDNA amplification

Live snails were washed in sterilised water, and then their shells were cleaned with 70% ethanol and removed. Whole snails were dissected individually on glass slides under a microscope, and those confirmed to have no helminthic infection were used for further study. For each species of snail, the whole soft body of each snail without the intestinal tract was carefully washed three times with ice cold RNAase-free 0.3% NaCl and then pooled and stored immediately in liquid nitrogen.

The total RNA from each sample was extracted using TRIzol Reagent (Ambion, Carlsbad, CA), in accordance with the manufacturer's instructions. The integrity and size distribution of the RNA was evaluated using agarose gel electrophoresis, and the RNA concentration determined using a NanoDrop 2000 spectrophotometer. Finally, single-stranded cDNAs for each sample were synthesised using a PrimeScript RT Reagent Kit with gDNA Eraser (Takara, Tokyo, Japan).

A partial cDNA fragment of *OhAOX* was amplified from *O. hupensis* cDNA using the following specific primers: OhAOX-F, 5′-TGAACTCCAAGCAGAGCGAAT -3′; OhAOX-R, 5′-TGCTGGTTGGGATGAAAACTG-3′. These were designed based on the *AOX* reference sequence inferred from transcriptomics. Partial cDNA fragments of *BgAOX*, *BaAOX*, and *BsAOX* were amplified from the corresponding snail cDNA with the following specific primers: Bg/Ba/BsAOX-F, 5′-ATGAACAGGGTTTCAATTATTCGAG-3′; Bg/Ba/BsAOX-R, 5′-TTAATGACCAGGTTTGTAAGG-3′. These were designed based on the predicted *B. glabrata AOX* (XM_013230806.1). Each polymerase chain reaction (PCR) was performed with 25 µL of 2× Phanta Max buffer (Vazyme Biotech, Nanjing, China), 2 µL cDNA template, 1 µL dNTP mix (10 mM each), 1 µL of each primer (10 μM), 1 µL Phanta Max Super-Fidelity DNA polymerase (Vazyme Biotech), and water to a final volume of 100 µl. Amplification was performed under the following conditions: 95 °C for 3 min; followed by 15 cycles of 30 s at 95 °C, 30 s at 62 °C (0.5 °C decrease per cycle), and 3 min at 72 °C; 15 cycles of 30 s at 95 °C, 30 s at 55 °C, and 3 min at 72 °C; and a final extension step at 72 °C for 10 min. Reactions were then checked by running 5 µL product on 1% agarose gel. The products of the successful amplifications were purified from the gel with AxyPrep DNA gel extraction kit (Axygen Biosciences, Union City, CA) and sent to Sangon Biotech (Shanghai, China) for sequencing.

The partial sequences of *OhAOX*, *BgAOX*, *BaAOX*, and *BsAOX* were blasted with the reference sequences and other mollusc AOXs (https://blast.ncbi.nlm.nih.gov/Blast.cgi?PROGRAM=blastn&PAGE_TYPE=BlastSearch&LINK_LOC=blasthome). Based on the verified 2,909 base pair (bp) partial cDNA of *OhAOX* and 1,023 bp partial cDNA of *Bg/Ba/BsAOX*, gene-specific primers in RACE-PCR and specific primers (SP) in genome walking were designed (for details about the primers, see Additional file 1: Table S1). The 5′- and 3′-RACE-Ready cDNAs were synthesised from the total RNA of the corresponding snail by using a Simple Modular Architecture Research Tool (SMART) RACE cDNA Amplification Kit (Clontech, Mountain View, CA). The RACE procedure was performed with touchdown PCR following the manufacturer’s instructions, and amplified products were purified from gels and sequenced. Geneious V8.1.8 [31] was used to complete and identify the combined cDNA sequences based on each partial fragment. BLAST analysis was used to check each combined sequence to ensure complete and unique.

#### Sequence features, protein structure, and phylogenetic analysis

BLASTP (https://blast.ncbi.nlm.nih.gov/Blast.cgi?PROGRAM=blastp&PAGE_TYPE=BlastSearch&LINK_LOC=blasthome

) was used to confirm the high similarity (37.62–75.42%) between OhAOX/BgAOX/BsAOX/BaAOX and other AOXs from 63 species, including dicotyledonous plants, monocotyledonous plants, fungi, protists, and molluscs (for species, see Additional file 2: Table S2). After alignment in ClustalW (https://www.genome.jp/tools-bin/clustalw) with protein sequences, the .aln file was uploaded to ESPrit3.0 (https://espript.ibcp.fr/ESPript/cgi-bin/ESPript.cgi), to predict the secondary structure of OhAOX, BgAOX, BaAOX, and BsAOX using the *Trypanosoma brucei* AOX (TbAOX) protein structure as a template. Also, the conserved region analysis, amino acid residue analysis, and post-translational regulation prediction of OhAOX, BgAOX, BaAOX, and BsAOX were conducted by aligning the proteins with that of TbAOX. The best model for the tertiary structure of OhAOX, BgAOX, BaAOX, and BsAOX was evaluated in SWISS-MODEL (Expert Protein Analysis System; https://swissmodel.expasy.org/) with Global Model Quality Estimation and Qualitative Model Energy Analysis.

The complete protein sequences of OhAOX, BgAOX, BaAOX, and BsAOX were aligned using ClustalW, together with those of another 65 AOXs (Additional file 2: Table S2). Thirty of these AOXs were from animals: six species of the phylum Mollusca, eight species of the phylum Cnidaria, four species of the phylum Echinodermata, four species of the phylum Euglenozoa [32–34], one species of the phylum Chordata, two species of the phylum Apicomplexa, one species of the phylum Ciliophora [35], one species of the phylum Choanozoa, and one species of the phylum Brachiopoda. Twenty were from plants: 17 species of the phylum Streptophyta [36–42], and three of the phylum Chlorophyta. Twelve of the AOXs were from fungi: five species of the phylum Basidiomycota and seven of the phylum Ascomycota. Three of the AOXs were from bacterial species of the phylum Proteobacteria. The protein sequences of ribonucleotide reductase from *B. glabrata* (XP_013082032.1) and *O. hupensis* (obtained from previous transcriptomic data [18]) were set as outgroups. The phylogenetic trees were then constructed using the maximum likelihood method in Molecular Evolutionary Genetics Analysis (MEGA)X [43] with 1000 bootstrap replicates. The LG + G evolutionary model was the model with the best fit for protein sequence evolution for AOXs, with the default settings based on the Bayesian information criterion.

### Niclosamide treatment and collection of specific tissues from *O. hupensis*

In accordance with previous molluscicidal assays [14, 17, 18], niclosamide was used at a final concentration of 0.1 mg/L. The solution was prepared with 50% wettable powder of niclosamide (WPN) (production license HNP 32280-N0013, product standard no. Q/320623 PAJ 004-2019; Luosen Chemical, Jiangsu, China). Twenty active, mature *O. hupensis* snails were individually exposed to 80 mL WPN solution, or H_2_O, in 100-mL flasks. After being separately immersed for 6, 12, 24, and 48 h, the snails were washed with dechlorinated water, and mortality assessed by the mobility of the head and foot muscle (head–foot) under the microscope [44]. All the treatments were performed in parallel with from four to six replicates at each time point, and repeated three times. Mortality data were statistically analysed using ANOVA (SPSS 20.0).

The live snails of each group were dissected individually under a microscope. For RNA and protein extraction, the whole soft bodies were cleaned and stored in liquid nitrogen. In addition, some snails were dissected for the separate collection of the head-foot region and liver-gonad. For histochemical observations, the whole soft body, head-foot region, and liver-gonad of each group were separately fixed in 4% polyformaldehyde and embedded in paraffin. Some of these parts were individually embedded with optimum cutting temperature compound (Sakura, Torrance, USA), snap-frozen in liquid nitrogen, and stored at − 80 °C.

### Dynamic quantification and tissue localisation of OhAOX mRNA, and protein levels after WPN treatment

#### Expression profile of OhAOX in different tissues determined by quantitative real-time PCR

At time points from 0 to 48 h after WPN treatment, the single-stranded cDNAs from the different tissues, including the whole soft body, head-foot region, and liver-gonad, were subjected to random-hexamer priming and synthesised from the corresponding extracted RNA using a PrimeScript RT Reagent Kit with gDNA Eraser (Takara). Before the quantitative real-time PCR (qPCR) was carried out, semi-quantitative PCR was performed to confirm the presence of the *OhAOX* mRNA in the tissue and the specificity of the primers in the amplification (for primers, see Additional file 3: Table S3). Specific primers were synthesised based on the verified *OhAOX* sequence. At the same time, a partial sequence of the 18S ribosomal RNA gene of *O. hupensis* (AF367667) was also used to monitor the transcription as an internal control for normalisation. For each sample, qPCR was performed in triplicate in a 20-μL reaction volume, using TB Green Premix Ex Taq II (Tli RNaseH Plus; Takara) and a Bio-Rad CFX96 thermal cycler, with the following protocol: 94 °C for 40 s, followed by 40 cycles of 94 °C for 5 s, 60 °C for 40 s, and 72 °C for 20 s, and melt curves analysis at 95 °C for 15 s, 65 °C for 10 s, and 95 °C for 10 s. The expression profiles of *OhAOX* in the different tissues after WPN treatment were normalised to the expression of the 18S gene using the comparative delta-delta Ct method [45] after the amplification efficiency was adjusted to 1.87–2.26. The results were statistically analysed for at least six samples per group using one-way ANOVA (among groups) or independent* t*-test analysis (between groups) in SPSS 20.0.

#### Localisation of OhAOX in tissues by in situ hybridisation

The head-foot and liver-gonad sections embedded and frozen with optimum cutting temperature compound were collected at 0–48 h post-treatment and cut into 5- to 8-μm-thick slices for in situ hybridisation with the 5'-digoxigenin-labelled RNA probes (5′-ACATACCAGATGCTGAACAGGGTCACAAACACAC-3′), to observe the localisation of *OhAOX*. Each slide was fixed in RNAase-free 4% polyformaldehyde for 10 min, followed by three washes with phosphate-buffered saline (PBS) for 5 min, and then incubated in 20 µg/mL proteinase K for 10 min at 37 °C for antigen retrieval. After incubation in pre-hybridisation solution at 37 °C for 1 h, the diluted probes (200 ng/mL) were added to the slides for hybridisation at 42 ℃ overnight, followed by stringent washing with saline sodium citrate and blocking with 8% rabbit serum (Roche, Basel, Switzerland) at room temperature for 30 min. The blocking buffer was then drained off, the diluted (1:400) anti-digoxigenin-AP (Jackson, Lancaster, USA) was added, and the samples incubated at 37 ℃ for 50 min. After applying Tris-buffered saline (TBS) washes, 5-bromo-4-chloro-3-indolyl phosphate/nitro blue tetrazolium was added to each slide; the slides were incubated for an optimal time to develop a purple-blue colour, then rinsed in distilled water, air dried, and sealed. The slides were observed under an Olympus CX431 microscope. The entire area of tissue on each slide was photographed under 100× magnification. The integrated optical density (IOD) and area were calculated for each photo with Image Pro-Plus [46] to quantify the *OhAOX* mRNA in the different tissues. Total IOD/total area was calculated for each slide, and the staining data statistically assessed using one-way ANOVA (among groups) or independent* t*-test analysis (between groups) in SPSS 20.0.

#### Expression of recombinant OhAOX protein and specificity of anti-OhAOX serum

A pair of primers that included* Sal*I and* Not*I restriction sites and protective bases were designed based on the verified complete cDNA sequence of *OhAOX*, the forward primer 5′-GCGTCGACTCATGTCGTTGTGTTTTAGTGCGT-3′ and the reverse primer 5′-ATAAGAATGCGGCCGCCTGTCCAGGCTTGTAAGGGTTA-3′. PCR was performed to amplify the complete open reading frame (ORF) of *OhAOX* from *O. hupensis* cDNA, and the purified PCR product was digested and ligated into pGEX-4 T-1 plasmid DNA. The resulting pGEX-4 T-1-*OhAOX* plasmid was then transformed into BL21 (DE3) competent cells, and positive clones were selected by PCR and sequencing. The expression of the recombinant protein was induced by adding isopropyl-β-thiogalactopyranoside to the culture medium to a final concentration of 0.2 mM and the expressed protein was purified using glutathione agarose beads (Smart-Lifesciences, Changzhou, China). After successful cleavage with thrombin, affinity GST tags were separated from the recombinant protein, and recombinant OhAOX protein was further purified with sodium dodecyl sulfate–polyacrylamide gel electrophoresis (SDS-PAGE), gel extraction and dialysis recycling. The concentration of extracted purified OhAOX protein was calculated by the bicinchoninic acid protein assay.

An antiserum to the recombinant OhAOX protein was generated by subcutaneous injection of five laboratory Kunming mice with 30 µg purified OhAOX protein per mouse in the back and abdomen, followed by three boosts with the same dose of protein at 2-week intervals. The mice were sacrificed, and sera were collected after the antiserum titer was confirmed by enzyme-linked immunosorbent assay.

The total protein extracted from the whole soft body of *O. hupensis* by an SDS lysis method (Beyotime, Shanghai, China) was screened by western blot to evaluate the specificity of the anti-OhAOX serum. Briefly, 60 µg of snail crude extract was loaded and separated by SDS-PAGE, and 0.2 µg purified recombinant OhAOX protein was loaded at the same time as the positive control. The separated proteins in the gel were then electrophoretically transferred to a polyvinylidene fluoride membrane at 200 mA for 2 h at 4 °C, and then blocked with 5% skim milk in Tris-buffered saline containing 0.05% TWEEN 20 for 2 h. Next, the membrane was probed with 1:300 diluted anti-OhAOX serum or PBS immune serum (primary antibody) at 4 °C overnight. After washing three times with PBS containing 0.05% Tween 20, the membrane was probed with 1:10,000 diluted horseradish peroxidase (HRP)-conjugated anti-mouse immunoglobulin G (IgG) (ABclonal, Wuhan, China) (secondary antibody) in PBS containing 0.05% Tween 20 for 2 h. The membrane was then washed three times and visualised using an enhanced chemiluminescence kit (Epizyme, Shanghai, China). Immunoblot images were acquired with the G:Box Chemi Imaging System analyser (Labgene, Châtel-Saint Denis, Switzerland).

#### Dynamic expression profile of OhAOX protein by western blot

The dynamic expression profiles of OhAOX protein in *O. hupensis* snails were evaluated by a semi-quantified western blot. Briefly, the frozen soft bodies of the snails at 0–48 h post-treatment were homogenised separately in SDS lysis buffer (Beyotime, Shanghai, China), and 100 µg of extracted protein of each sample was electrophoresed and transferred to a polyvinylidene fluoride membrane. The membrane was blocked with 5% skim milk, incubated with 1:500 diluted mice serum, then washed and incubated with 1:10,000 diluted HRP-conjugated anti-mouse IgG. α-Tubulin was set as the control by using 1:10,000 diluted anti-α-tubulin monoclonal antibody (Proteintech, Manchester, UK) as the first antibody. After visualisation using the enhanced chemiluminescence method, the grey values of OhAOX-positive and α-tubulin-positive bands were analysed in Image J. The relative quantification of OhAOX protein was calculated using the grey values, which were calibrated using the α-tubulin grey value as the internal control. The statistical analysis of the quantity of OhAOX protein among the different groups was performed using one-way ANOVA in SPSS20.0

#### Immunohistochemical localisation of OhAOX

The tissue localisation of OhAOX protein was performed using immunohistochemical staining. Paraffin sections (3 µm) of head-foot and liver-gonad were treated with xylene (two steps of 100% for 5 min for each) and rehydrated in an alcohol gradient (100%, 100%, 95%, 80%, and 70%) and PBS. After heat-induced epitope retrieval and blocking with 10% sheep serum (Roche, Basel, Switzerland) for 1 h at 37 ℃, the slides were incubated with 1:50 diluted mouse anti-OhAOX serum, PBS immune serum, and blocking sheep serum, respectively, at 4 ℃ overnight. The slides were washed three times for 5 min per wash in PBS and incubated for 1 h at 37 ℃ with 1:100 diluted HRP-conjugated goat anti-mouse IgG (ABclonal, Wuhan, China). Immunoreactivity was developed using 3,3′-diaminobenzidine. The slides were washed in tap water and counterstained with haematoxylin, then dehydrated in ethanol and hyalinised in xylene, air dried, and sealed. The sections were examined and photographed using an Olympus light microscope, and the brown-yellow regions were identified as OhAOX positive. Five visually different areas were randomly selected for each slide at a magnification of 400×, and the IOD and area of each image were measured using Image Pro-Plus [46]. Total IOD (sum)/total area (sum) was used for the quantitative analysis of OhAOX protein among groups, and the statistical analysis was performed using one-way ANOVA in SPSS 20.0.

In addition, the same sections as used above were also stained using the conventional hematoxylin–eosin method as the reference for the immunohistochemical staining to observe the positive sites and cell types.

### Expression profile of genes in the mitochondrial respiratory complex, and the production of ROS in snails after WPN treatment

#### Expression profile of genes in the mitochondrial respiratory complex determined by qPCR

The single-stranded cDNAs from the whole soft body at 0–48 h post-treatment were prepared from the same sample used for *OhAOX* qPCR. To assess the response of other genes in the mitochondrial respiratory pathway, the expression profiles of several genes, including those encoding nicotinamide adenine dinucleotide (NADH) dehydrogenase, SDH, cytochrome c reductase (CCR), CCO, and ATP synthase, were investigated by qPCR using the same procedure and system as used for *OhAOX*. Primers were designed based on previously determined transcriptomic sequences [18] (for primers, see Additional file 3: Table S3). The expression profile of each target gene was normalised to the expression of the 18S gene using the comparative delta-delta Ct method [45] after the amplification efficiency of the genes was adjusted to 1.87–2.38. The results were statistically analysed using one-way ANOVA (among groups) or independent* t*-test analysis (between groups) in SPSS 20.0 software.

#### ROS production

The fresh soft body, head-foot region, and liver-gonad of the snails at 0–48 h post-treatment were collected individually, five biological replicates were prepared for the same tissue at each time point and the experiment repeated three times. To each sample was added 150 µL PBS, followed by phenylmethylsulfonyl fluoride to a final concentration of 0.5 mM; the mixture was then ground on ice, and centrifuged at 12,000* g* at 4 ℃ for 20 min. The supernatant was collected, and the amount of extracted protein was calibrated using bovine serum albumin (BSA).

The 10–15 µL volume of supernatant per individual sample (10 µL for a liver-gonad sample and 15 µL for a soft body or head-foot sample) and 60–75 µL PBS (60 µL for a soft body sample, 65 µL for a liver-gonad sample, and 75 µL for a head-foot sample) were added to each well of the black 96-well plate. The 1:50 diluted 2',7'-dichlorodihydrofluorescein diacetate (DCFH-DA) in PBS (10 µL for a head-foot sample and 25 µL for a soft body or liver-gonad sample) was added to each well to a final concentration of 25 µM, followed by incubation at 37 ℃ for 30 min in the dark. The intensity of DCF fluorescence was evaluated at 488 nm and 525 nm to indicate oxidative stress. The ROS in each sample was calibrated using the amount the individual extracted protein and statistically analysed among the different groups using one-way ANOVA.

### Molluscicidal assay with niclosamide and AOX inhibitor

The qPCR showed that the quantity of *OhAOX* in the snails had increased significantly by as early as 6 h after WPN treatment. In this assay, salicylhydroxamic acid (SHAM) (CAS, 89-73-6; product no. S817724; Shanghai Macklin Biochemical, China), a specific inhibitor of AOX enzyme activity, was added together with WPN at 0 or at 6 h to observe the inhibition of the molluscicidal effect of niclosamide by AOX. Using the same experimental design as described above for the niclosamide treatment, the snails were randomly divided into the following groups: WPN group, H_2_O group, methanol group (control solvent for SHAM), SHAM 0 h group (SHAM was added at 0 h), SHAM 6 h group (SHAM was added at 6 h), WPN + SHAM 0 h group (SHAM was added at 0 h post-WPN treatment), and WPN + SHAM 6 h group (SHAM was added at 6 h post-WPN treatment). The final concentrations of WPN and SHAM were 0.1 mg/L and 0.4 mM, respectively, and the concentration of methanol (in the methanol group and the SHAM-containing groups) was 0.08%. Three biological replicates were set up in parallel for each group. All treatments were performed in triplicate. Snail mortality was calculated as described above, at 0, 6, 12, 24, and 48 h after treatment, and the data statistically analysed with the chi-square test and the chi-square test for Trend in SPSS 20.0.

The results of this assay suggested that SHAM could significantly enhance the molluscicidal effect of niclosamide. The dosage of WPN was reduced in the following experiment to investigate the potential ability of AOX to diminish the molluscicidal effect of niclosamide. With the same experimental design as described above for the niclosamide treatment, the snails were randomly divided into 11 groups: H_2_O group, SHAM group, methanol group, 0.1 mg/L WPN group, 0.08 mg/L WPN group, 0.06 mg/L WPN group, 0.04 mg/L WPN group, 0.1 mg/L WPN + SHAM group, 0.08 mg/L WPN + SHAM group, 0.06 mg/L WPN + SHAM group, 0.04 mg/L WPN + SHAM group. The final concentration of SHAM in all relevant group was 0.4 mM, and the solutions were added in each group at 0 h. All the treatments were performed in triplicate. Snail mortality was calculated for each group at 0, 6, 12, 24, and 48 h after treatment and the data statistically analysed with the chi-square test and a chi-square test for Trend in SPSS 20.0.

## Results

### Identification and sequence characteristics of snail* AOX*s

* OhAOX* cDNA was found to comprise 4412 bp through cDNA amplification and sequencing, with a 1266-bp complete ORF, but varied with respect to a six base (CTAAAC) deletion encoding 419 or 421 amino acids (aa). The identified cDNAs of *BgAOX*, *BaAOX* and *BsAOX* were 2003 bp, 1974 bp, and 1238 bp in length, with complete ORFs of 1023 bp, 1029 bp, and 1029 bp encoding 340 aa, 342 aa, and 342 aa, respectively. These confirmed *AOX*s were deposited in GenBank under accession numbers MZ436164-MZ436167. The *OhAOX* cDNA had the highest similarity, 74.65%, with the predicted *Branchiostoma belcheri AOX* mRNA (XM_019758882.1). *BgAOX*, *BaAOX*, and *BsAOX* had the highest similarity, 94.69–97.41%, with the predicted *B. glabrata AOX* transcript variant X1 (XM_013230806.1), derived from the *B. glabrata* genomic sequence. Identity between the predicted OhAOX protein sequence and those of BgAOX, BaAOX, and BsAOX was 63.12%, 57.35%, and 57.65%, respectively.

All four AOXs were identified as members of the ferritin-like superfamily through Conserved Domain Search and SMART analysis, with conserved motifs located at 194–408 aa for OhAOX, 113–327 aa for BgAOX, and 115–329 aa for BaAOX and BsAOX (Additional file 4: Fig. S1). OhAOX contained two transmembrane helixes (TMhelix) located at 248–270 aa and 306–328 aa, while none of the three *Biomphalaria* AOXs had a TMhelix (Additional file 4: Fig. S1). The four AOXs had no signal peptide and were deduced to be unstable proteins with a high aliphatic index (77.60–80.67). Ten α-helices and six conserved diiron-binding motifs were identified in each AOX, as in TbAOX (Additional file 5: Fig. S2). The NP-[YF]-XPG-[KQE] motif, an animal AOX-specific motif, was identified in the C-terminal of the four snail AOXs (Additional file 5: Fig. S2).

The tertiary structure of TbAOX was used as the template to build the homology model, in which OhAOX, BgAOX, BaAOX, and BsAOX showed sequence identity of 49.58%, 49.57%, 49.36%, and 48.94%, respectively, with Global Model Quality Estimation values of 0.41, 0.50, 0.50, and 0.50, and Qualitative Model Energy Analysis values of − 3.89, − 2.62, − 2.67, and − 2.61, respectively. Similar to TbAOX, each snail AOX was identified as a homodimer, with a diiron core in each monomer surrounded by a four-helix bundle and ligated by four glutamic acid and two histidine residues (Additional file 6: Fig. S3), which were considered the active sites of the AOX.

### Phylogenetic analysis of snail AOXs

**Fig. 1:**
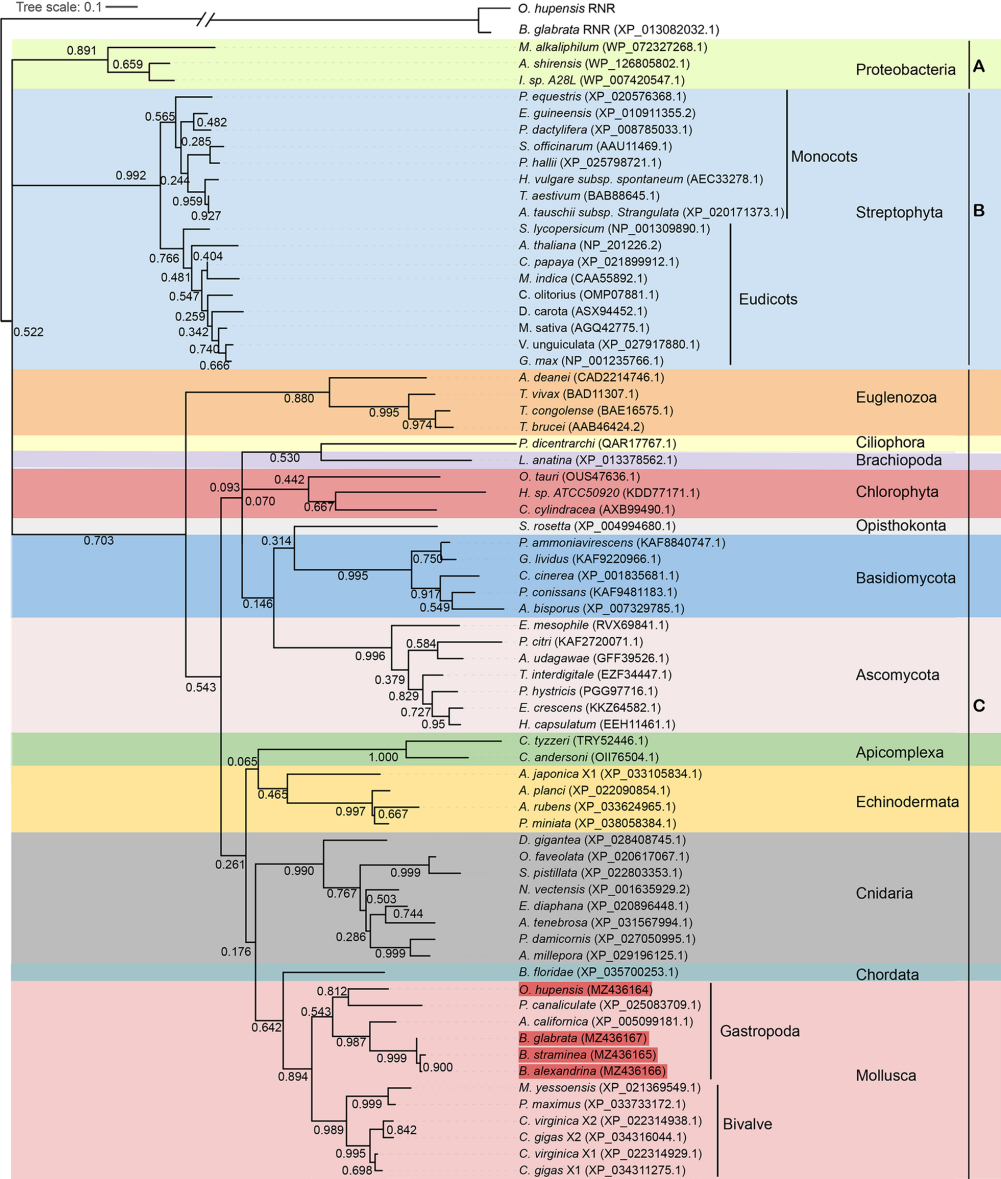
Phylogenetic tree (maximum likelihood; ML) of alternative oxidases (AOXs) based on protein sequences. The number beside each node shows the ML support rate (bootstrap value). The four snail AOXs identified in this study are highlighted in red (in the Gastropoda branch)

Sixty-nine AOXs were separated into three main clades (Fig. 1). Clade A was a bacterial AOX group consisting of AOXs from three species in the phylum Proteobacteria. Clade B was a Viridiplantae group containing all AOXs from 17 species of Streptophyta, with a high supporting rate but not including the Chlorophyta AOX. Clade B was further separated into monocot and eudicot groups (Fig. 1). The other 49 AOXs from metazoans, protists, fungae, and chlorophytes were clustered into the giant clade C. All AOXs in clade C clustered together well at the level of phylum, with high supporting rates (Fig. 1). The Mollusca group diverged distinctly into the Gastropoda and Bivalvia AOX branches. The AOX of *O. hupensis*, which is in the order Littorinimorpha, clustered closely together with the AOX of *P. canaliculate*, which is in the Architaenioglossa, while the three AOXs of the *Biomphalaria*, which are in the order Basommatophora, clustered closely together with the AOX of *Aplysia californica*, a species in the family Aplysiidae. Interestingly, the Mollusca AOX group first clustered with the AOX of *B. floridae*, which is in the phylum Chordata, and then with the Apicomplexa AOX to form one of the main clades in the Metazoa. It is worth mentioning that the AOX of *Lingula anatina*, of the metazoan phylum Brachiopoda, did not cluster together with other metazoan AOXs but clustered closely with the AOX of *Philasterides dicentrarchi*, which is in the phylum Ciliophora, kingdom Protista, and then with the AOX of Chlorophyta, which is in the kingdom Viridiplantae. In addition, the AOX of the protist *Salpingoeca rosetta*, which is in the phylum Choanozoa, clustered with the AOXs of the fungal phylum Basidiomycota at a low support rate, and then with the AOXs of Ascomycota to form the main fungal group.

### Dynamic expression and localisation of *OhAOX* mRNA and OhAOX protein after WPN treatment

#### Up-regulation of* OhAOX* mRNA and OhAOX protein

**Fig. 2a–c:**
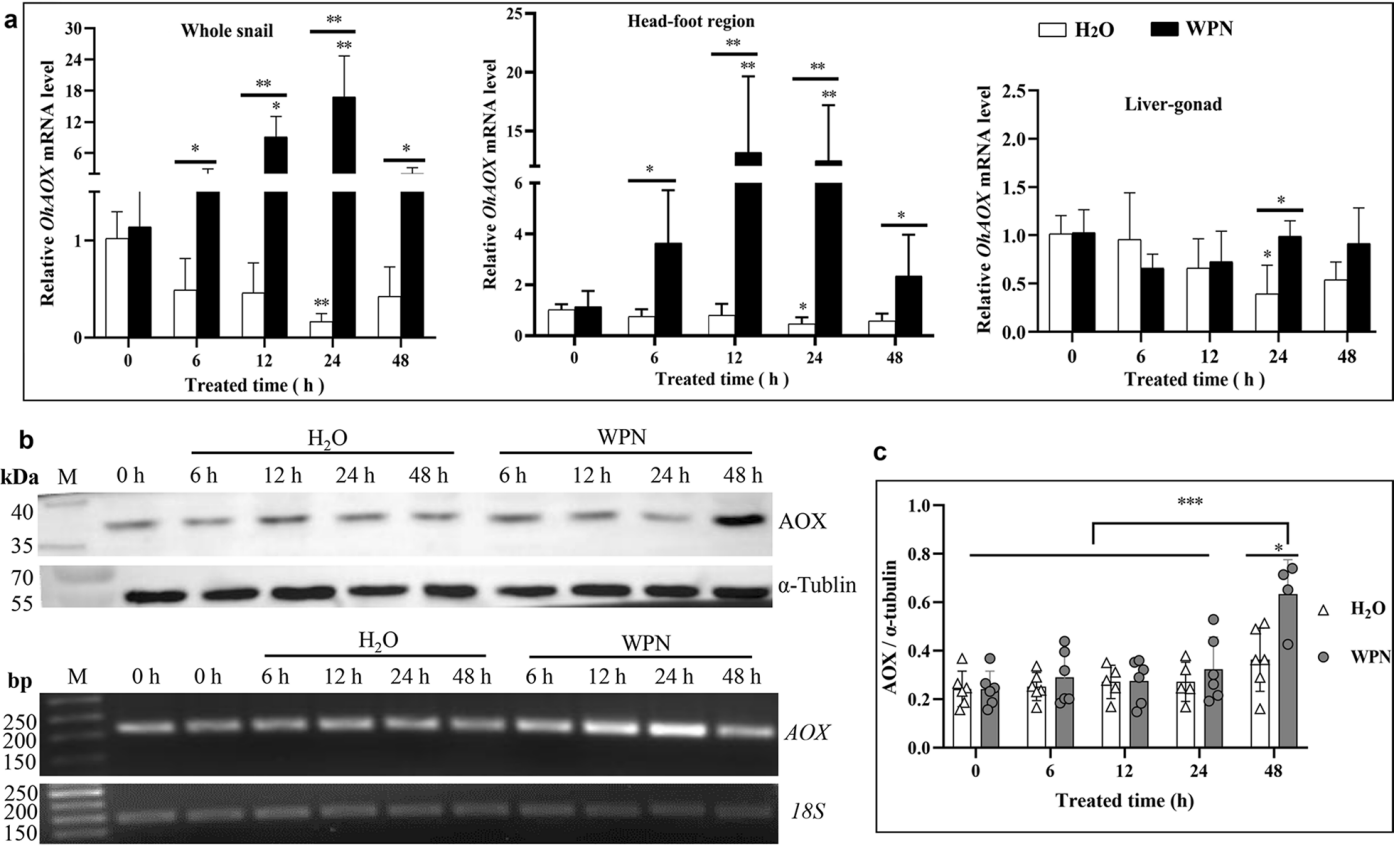
Dynamic expression profiles of *Oncomelania hupensis AOX* (*OhAOX*) messenger RNA (*mRNA*) and OhAOX protein in *O. hupensis* snails under wettable powder of niclosamide (*WPN*) stress. **a** Expression profiles of *OhAOX* in the whole snail, head and foot muscle (*head-foot*) region, and liver and gonad (*liver-gonad*) region after WPN treatment. Asterisks indicate significant difference (*P < 0.05, **P < 0.01) between time points and 0 h within the same treatment; underlined asterisks indicate significant difference between WPN- and H_2_O-treated snails at the same time point. **b** Expression of OhAOX protein (upper panel) in the whole snail 0–48 h after WPN and H_2_O treatment. The *OhAOX* mRNA profile calculated using quantitative real-time PCR (qPCR) is shown in the lower panel (and corresponds with the whole snail data in **a**). **c** Statistical analysis of OhAOX protein expression in the western blot (**b**); *P < 0.05, ***P < 0.001.* bp* Base pairs

The quantity of *OhAOX* mRNA in the whole snail increased immediately and thence continuously after WPN treatment (Fig. 2a), reaching 8.04 times its value at 0 h after 12 h (*P* < 0.01). The highest increase was recorded at 24 h (14.75 times that at 0 h, *P* < 0.01), after which the level dropped, and at 48 h was almost the same as at 0 h (1.44 times that at 0 h,* P* > 0.05). Meanwhile, *OhAOX* mRNA expression decreased continuously from 0 h in H_2_O-treated snails, and was significantly lower at 24 h post-treatment (0.16 times that at 0 h, *P* < 0.05) (Fig. 2a). Moreover, the quantity of *OhAOX* mRNA in WPN-treated snails was significantly higher than that in H_2_O-treated snails at all time points, i.e. 102.39 times higher at 24 h (*P* < 0.01), at which point it had reached its highest level, and 3.89 times higher at 48 h (*P* < 0.05) (Fig. 2a).

The dynamic expression profiles of *OhAOX* mRNA in the different tissues, including those of the head-foot region and liver-gonad, were also calculated 0–48 h after WPN treatment. *OhAOX* mRNA in the head-foot region showed a similar trend to that of the whole snail. It increased after WPN treatment and reached its highest level at 12 h (11.52 times that at 0 h, *P* < 0.01), then decreased gradually until 48 h, at which point it had returned to a level closer to that considered normal (2.06 times that at 0 h, *P* > 0.05). For the snails that had been immersed in H_2_O, *OhAOX* mRNA in the head-foot region decreased slightly, showing a lower level at 24 h (0.46 times that at 0 h, *P* < 0.05) (Fig. 2a). In summary, *OhAOX* mRNA in the head-foot region was significantly higher after WPN treatment (4.86–26.87 times greater, *P* < 0.05 or 0.01) than after H_2_O treatment, at each time point (Fig. 2a). The expression of *OhAOX* mRNA in the liver-gonad of snails under WPN stress differed from that in the whole snail and the head-foot region, as no significant difference was observed from 0 to 48 h (*P* > 0.05) (Fig. 2a). The expression of *OhAOX* mRNA in the liver-gonad showed no significant difference between the WPN and H_2_O treatments at most time points, except at 24 h, when it was 2.52 times higher in the WPN-treated snails than in the H_2_O-treated snails (*P* < 0.05) (Fig. 2a).

After the recombinant OhAOX protein had been purified, cleaved, and analysed using SDS-PAGE (Additional file 7: Fig. S4a, b, c), its mass was verified to be the expected 47 kDa. The existence and specificity of the polyclonal antibody in mouse anti-OhAOX serum were proven by a single band that reacted with the recombinant OhAOX protein, or crude protein, of the whole snail, in the western blot analysis (Additional file 7: Fig.  S4d). The natural OhAOX from the whole snail extract was smaller than the recombinant OhAOX protein (Additional file 7: Fig.  S4d), which could be explained by the transmembrane transfer of natural OhAOX from the nucleus to the mitochondrion.

The dynamic expression of the OhAOX protein in the snails after WPN treatment was further investigated and quantified using α-tubulin as the internal control (Fig. 2b). The intensity of the specific band showed that the expression of OhAOX protein increased slowly after WPN treatment, and that the highest level of the protein was reached at 48 h (Fig. 2b), which was statistically significantly higher than that at 0 h (2.61 times that at 0 h, *P* < 0.05) (Fig. 2c), while no significant difference was observed for snails 0–48 h after H_2_O treatment (Fig. 2b, c). It was evident that, under WPN stress, the increase of OhAOX protein was hysteretic to that of *OhAOX* mRNA (Fig. 2a, b), which increased to its highest level at 24 h after WPN treatment.

#### Tissue expression of* OhAOX* mRNA and OhAOX protein

**Fig. 3a, b:**
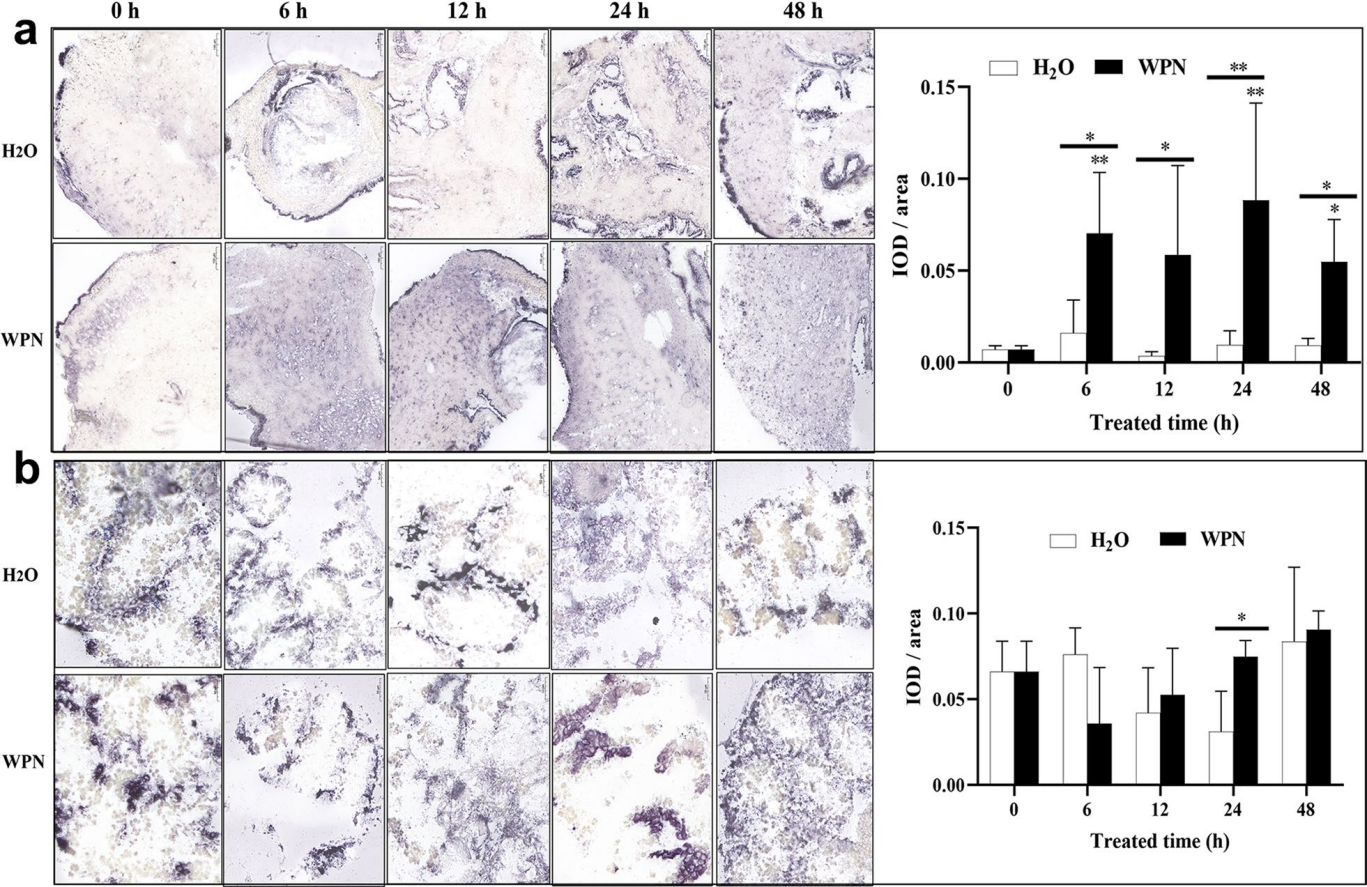
Tissue expression of *OhAOX* mRNA in *O. hupensis* after WPN treatment following in situ hybridisation. **a** Tissue distribution and statistical evaluation of *OhAOX* mRNA in the muscle of the head-foot region. **b** Tissue distribution and statistical evaluation of *OhAOX* mRNA in the liver. The purple-blue area was identified as a positive signal of *OhAOX* mRNA. Asterisks indicate statistically significant difference (*P < 0.05, **P < 0.01) in the quantity of *OhAOX* mRNA between the given time point and 0 h under the same treatment. Underlined asterisks indicate the level of significant difference between WPN- and H_2_O-treated groups at the same time point.* IOD* Integrated optical density; for other abbreviations, see Fig. 2

In untreated snails (0 h), the purple-blue colour indicating the presence of *OhAOX* mRNA was mainly distributed in the pellicle, liver, and the head-foot region, but was limited in the muscle (Additional file 8: Fig. S5); it also indicated positive expression in the testis. After WPN treatment, the head-foot muscle showed more distinct, positive signals at 6 h than at 0 h, and continued to show strong positive signals thereafter (Fig. 3a). The change in intensity of the positive signal showed a similar trend to the change in the expression profile of the protein in the qPCR, as it increased significantly 6 h after WPN treatment, when it was 9.99 times higher than that at 0 h, and was high from 12 to 48 h (7.69–13.33 times that at 0 h) (Fig. 3a). Meanwhile, in the H_2_O-treated snails, a positive signal only appeared sporadically in the head-foot region, and no significant difference was seen from 0 to 48 h (Fig. 3a). In summary, the positive signal of the head-foot under WPN stress was significantly stronger than that after H_2_O treatment at all time points from 6 to 48 h (Fig. 3a).

The positive signal of *OhAOX* mRNA in the liver tissue showed no significant difference (*P* < 0.05) from 0 to 48 h after WPN or H_2_O treatment (Fig. 3b), except at 24 h, when it was 2.40 times stronger in the WPN-treated snails than in the H_2_O-treated snails (Fig. 3b).

**Fig. 4a, b:**
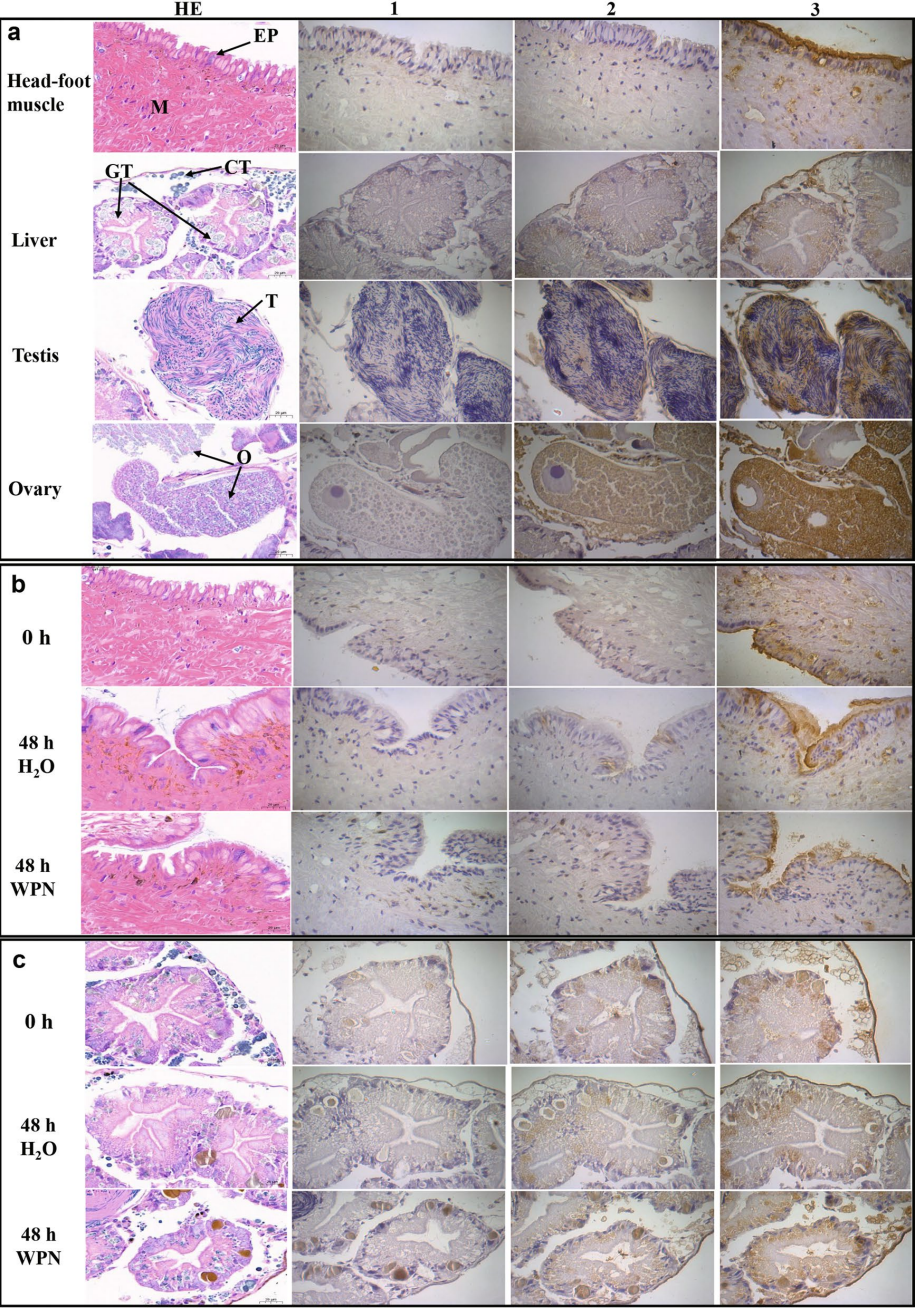
Tissue distribution of OhAOX protein determined by immunohistochemistry. **a** Different tissues of the untreated snails. **b** Head-foot muscle at 48 h after WPN treatment. **c** Liver at 48 h after WPN treatment. *1* Blank controls, *2* phosphate-buffered saline (PBS) immune serum, *3* anti-OhAOX serum,* HE* hematoxylin–eosin staining,* M* muscle,* EP* epithelium,* GT* gland tissue,* CT* connective tissue,* T* testis,* O* ovary. The regions stained brown-yellow indicate the distribution of OhAOX protein

The cellular localisation of OhAOX protein in different tissues was further investigated with an immunohistochemical assay. OhAOX protein was mainly located in the epithelium of the head-foot region, the reticular connective tissue in the liver-gonad, and the genital gland of untreated snails (0 h) (Fig. 4a). The positive signals in the gonads were mainly distributed in the testicular substrate and ovarian stroma, while there was no positive staining in the cytoplasm of immature and mature oocytes (Fig. 4a). In addition, a small amount of brown-yellow staining was observed in the muscle tissue of the head-foot region and the hepatic ducts (Fig. 4a). In accordance with the high level of OhAOX in snails at 48 h after WPN treatment in the western blot, the localisation of OhAOX protein at 48 h post-treatment was further investigated. The positive signals in the head-foot region were distributed mainly in the epithelium, with a few appearing in the muscle (Fig. 4b), but no significant difference (*P* > 0.05) was observed between 48 and 0 h in the H_2_O-treated or in the WPN-treated snails. Also, no significant difference was found in the head-foot region at 48 h between the H_2_O- and WPN-treated snails (Fig. 4b). In the liver, after WPN or H_2_O treatment, the positive signals were mainly observed in the same sites as those at 0 h, but the intensity increased significantly at 48 h after WPN treatment (*P* < 0.05) (Fig. 4c).

### Expression profiles of genes involved in the mitochondrial respiratory complex, and ROS production after WPN treatment of snails

**Fig. 5:**
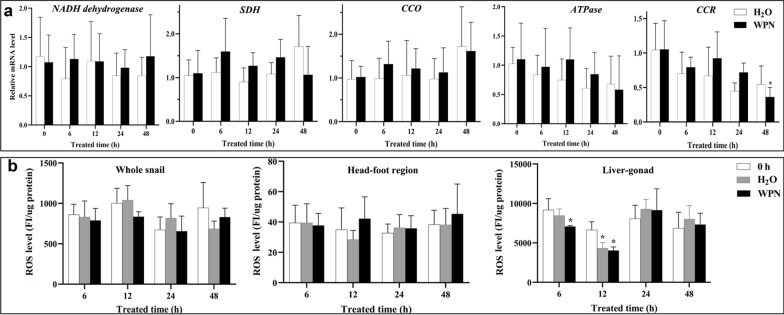
Expression profiles of genes involved in the mitochondrial respiratory complex (*MRC*), and reactive oxygen species (*ROS*) production in *O. hupensis* after WPN treatment. **a** Dynamic expression profile of genes coding for NADH dehydrogenase (*NADH*), succinic dehydrogenase (*SDH*), cytochrome C oxidase (*CCO*), ATPase, and cytochrome c reductase (*CCR*) in the whole snail determined using qPCR. **b** ROS production in the whole snail, head-foot region, and liver-gonad determined using the 2′,7′-dichlorodihydrofluorescein diacetate (DCFH-DA) method. Asterisk indicates statistically significant difference (*P < 0.05) when the level is compared with that at 0 h for the same tissue. For other abbreviations, see Fig. 2

For most of the genes involved in the mitochondrial respiratory complex (MRC) investigated here, including those encoding NADH dehydrogenase, SDH, CCO, and ATPase, no significant difference in the quantity of mRNA was observed between WPN-treated and H_2_O-treated snails at any time point. Also, no significant difference was recorded at any time point from 0 to 48 h within the same treatment (Fig. 5a). The expression profile of CCR mRNA had decreased to its lowest level (0.34 times that at 0 h) at 48 h after WPN treatment, which was significantly lower than that at 0 h (*P* < 0.05) (Fig. 5a). However, the expression of CCR in H_2_O-treated snails showed no difference from 0 to 48 h (*P* > 0.05).

The amount of ROS in the whole snail, head-foot region, and liver-gonad was calculated using the DCFH-DA assay. There was no significant difference in the level of ROS at the times points from 0 to 48 h (*P* > 0.05) in the whole snail and head-foot region after WPN or H_2_O treatment (Fig. 5b). Most of the ROS were produced in the liver-gonad (Fig. 5b), in which there was a significant decrease in levels at 6 h after WPN treatment (0.77 times that at 0 h, *P* < 0.05), and further decrease until ROS were at their lowest level at 12 h (0.61 times, *P* < 0.05). The levels had returned to normal at 24 h (1.07–1.14 times that at 0 h, *P* > 0.05) (Fig. 5b). The level of ROS in the liver-gonad fluctuated after H_2_O treatment, but no significant difference was shown for most time points (*P* > 0.05) when comparing the levels to that at 0 h, except at 12 h (0.65 times that at 0 h, *P* < 0.05) (Fig. 5b).

### Inhibition of OhAOX activity significantly enhanced the molluscicidal effect of niclosamide

**Fig. 6a, b:**
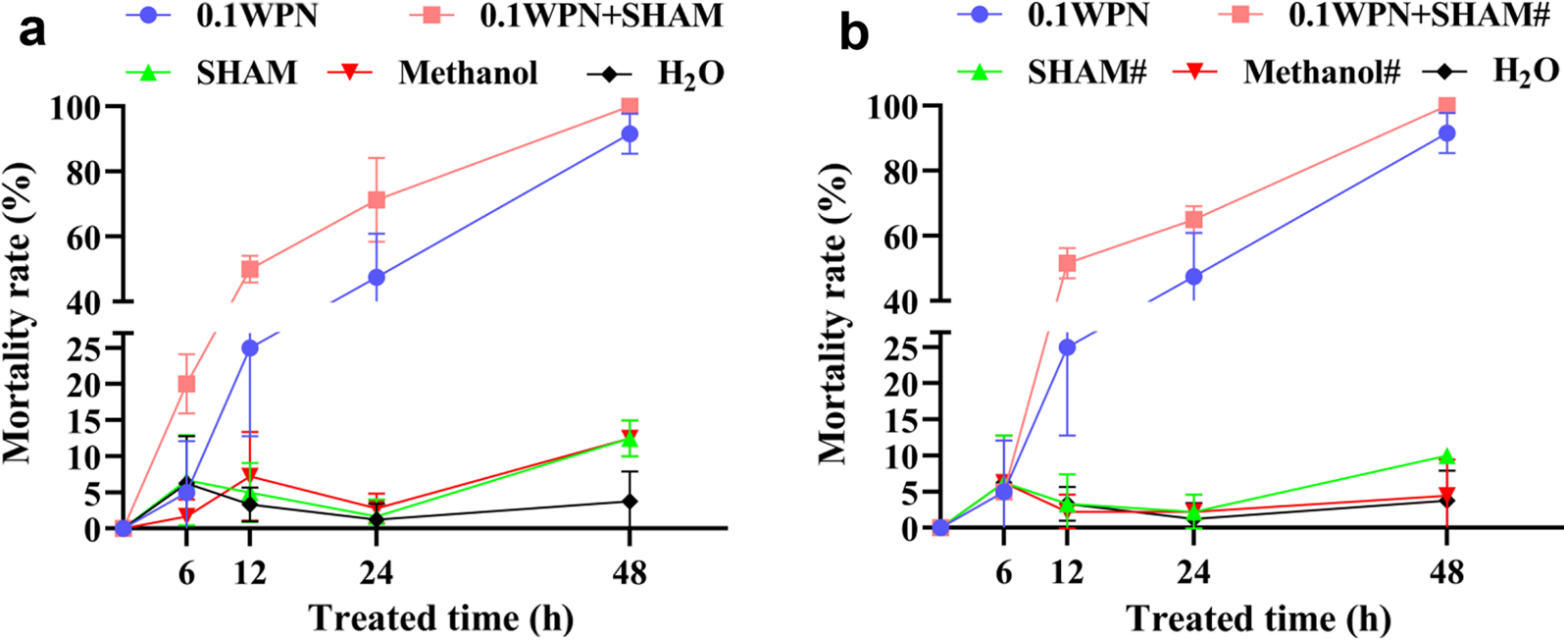
Dynamic mortality rate of *O. hupensis* after WPN treatment with the addition of salicylhydroxamic acid (*SHAM*). **a** SHAM added together with WPN at 0 h. **b** SHAM added at 6 h after WPN treatment.* 0.1WPN* Niclosamide at 0.1 mg/L,* SHAM* SHAM at 0.4 mM,* 0.1WPN–SHAM* niclosamide at 0.1 mg/L and SHAM at 0.4 mM (added at the same time),* Methanol* methanol at 0.08% (control solvent for SHAM), *hash symbol* indicates that the corresponding reagent was added at 6 h

The chi-square test for Trend was used to compare the mortality rates of snails, treated with the same solution, at different time points. The mortality rate of snails in the H_2_O group, SHAM group, methanol group, SHAM# group (where the hash symbol indicates that the reagent was added at 6 h), and methanol# group showed no significant difference from 6 to 48 h (*P* > 0.05) (Additional file 9: Table S4) (Fig. 6). In contrast, the mortality rates of snails in the WPN group, WPN + SHAM group, and WPN + SHAM# groups showed highly significant differences (*P* < 0.001) at time points from 6 to 48 h (Additional file 9: Table S4). The molluscicidal effect gradually increased with prolonged exposure of the snails to WPN (Fig. 6). Snail mortality significantly increased at 6 h in the WPN + SHAM group (by 20.00%, *P* < 0.05) (Fig. 6a) and at 12 h in the WPN group (by 25.00%, *P* < 0.05), and in the WPN + SHAM# group (by 51.67%, *P* < 0.05) (Additional file 9: Table S4) (Fig. 6b) compared to their mortality at 0 h (0.00%). In addition, the WPN + SHAM group (snails exposed to SHAM and WPN starting from 0 h) showed a highly significant mortality rate compared to the WPN group at every time point from 6 to 48 h (Additional file 9: Table S4) (Fig. 6a). Similarly, the WPN + SHAM# group (snails exposed to SHAM at 6 h after exposure to WPN) showed a significantly higher mortality rate than the WPN group at every time point after 12 h (*P* < 0.05) (Additional file 9: Table S4) (Fig. 6b).

**Fig. 7:**
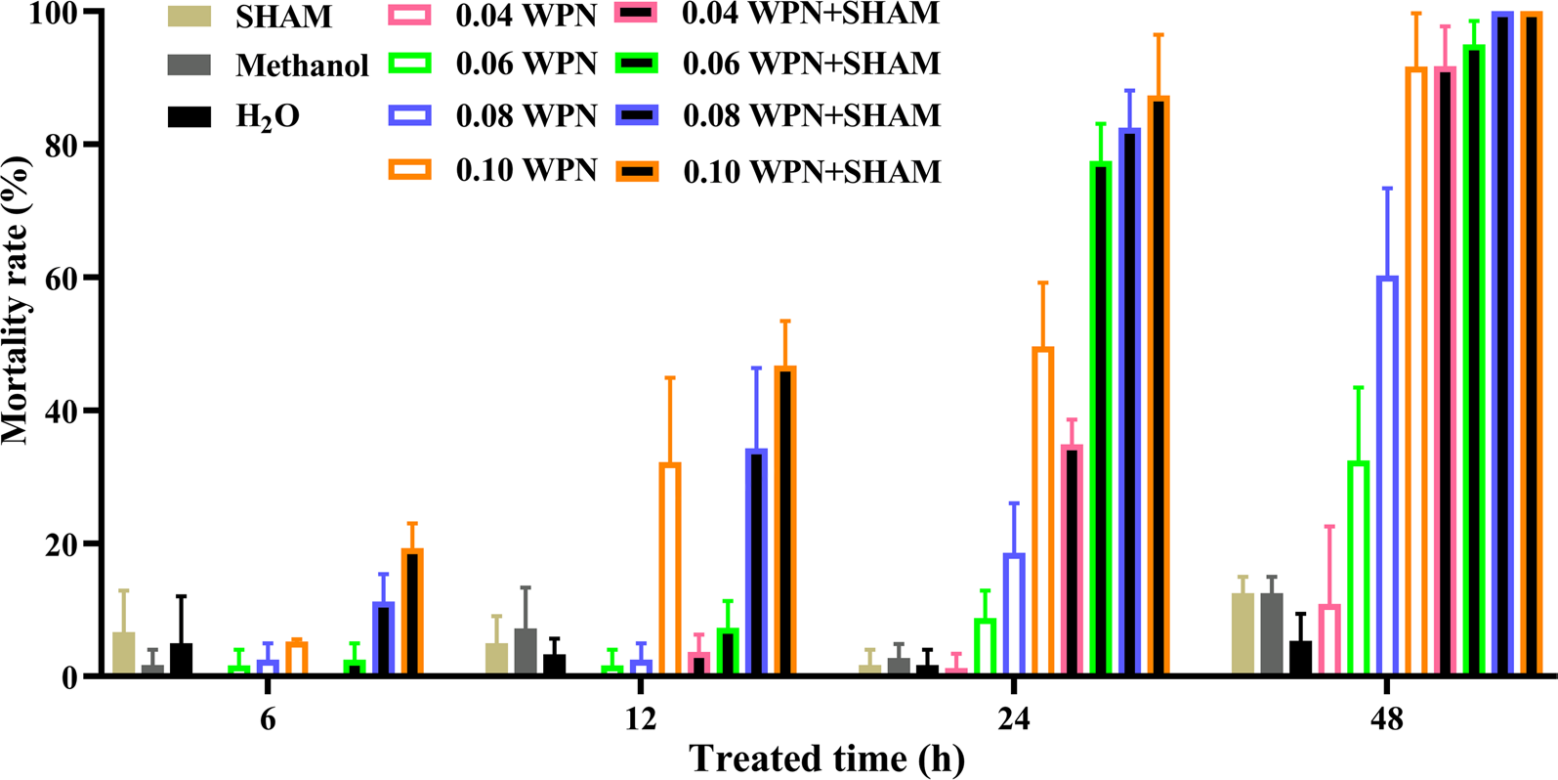
Mortality rate of *O. hupensis* treated with different concentrations of niclosamide with and without inhibition of OhAOX activity through the addition of SHAM. For abbreviations, see Figs. 2 and 6

Niclosamide was also applied at several lower concentrations together with SHAM at 0 h to evaluate the impact of AOX inhibition on the molluscicidal effect of niclosamide. The mortality rates of the 0.04–0.1 mg/L WPN groups and of the 0.04–0.1 mg/L WPN + SHAM groups were examined by a chi-square test for Trend. This showed that the molluscicidal effect increased with niclosamide concentration at all time points from 6 to 48 h, except for the WPN groups at 6 h (Table 1; Fig. 7).

**Table 1:** Mortality rate (%) of snails after wettable powder of niclosamide (*WPN*) treatment with or without the alternative oxidase (*AOX*) inhibitor salicylhydroxamic acid (*SHAM*)

Group	6 h	12 h	24 h	48 h	χ^2^	*P*
H_2_O	5.00 ± 7.07	3.33 ± 2.36	1.67 ± 2.36	5.33 ± 4.11	0.247	0.684
Methanol	1.67 ± 2.36	7.22 ± 6.14	2.78 ± 2.08	12.50 ± 2.50	3.761	0.056
SHAM	6.67 ± 6.24	5.00 ± 4.08	1.67 ± 2.36	12.50 ± 2.50	0.751	0.415
0.04 mg/L WPN	0.00 ± 0.00	0.00 ± 0.00	1.25 ± 2.17	10.89 ± 11.69 a, b	14.656	0.000
0.06 mg/L WPN	1.25 ± 2.17	1.25 ± 2.17	8.75 ± 4.15	32.50 ± 10.90* a, b, c	40.815	0.000
0.08 mg/L WPN	2.50 ± 2.50	2.50 ± 2.50	18.57 ± 7.49* a, b	60.24 ± 13.12* a, b, c	88.298	0.000
0.1 mg/L WPN	5.24 ± 0.34	32.21 ± 12.66* a	49.58 ± 9.60* a	91.67 ± 8.03* a, b, c	144.024	0.000
0.04 mg/L WPN + SHAM	0.00 ± 0.00	3.67 ± 2.62 d	34.90 ± 3.74* a, b	91.72 ± 6.02* a, b, c	216.473	0.000
0.06 mg/L WPN + SHAM	2.50 ± 2.50	7.32 ± 4.02 d	77.50 ± 5.59* a, b, d	95.00 ± 3.54* a, b, c	194.708	0.000
0.08 mg/L WPN + SHAM	11.25 ± 4.15	34.29 ± 12.05* a	82.50 ± 5.59* a, b, d	100.00 ± 0.00* a, b, c, d	161.253	0.000
0.1 mg/L WPN + SHAM	19.29 ± 3.75* d	46.68 ± 6.74* ad	87.32 ± 9.17* a, b, d	100.00 ± 0.00* a, b, c, d	189.074	0.000

Lowercase letters indicate the following: a, mortality at that time point was significantly higher than that at 6 h for the same group (*P* < 0.05); b, mortality at that time point was significantly higher than that at 12 h for the same group (*P* < 0.05); c, mortality at that time point was significantly higher than that at 24 h, for the same group (*P* < 0.05); d, significant difference in mortality between the treatment group and the 0.1 WPN group at that time point (*P* < 0.05)

* *P* < 0.05 (significant difference in mortality between that treatment group and the H_2_O group at that time point)

Compared to the H_2_O group, the mortality rate was significantly higher in the 0.1 mg/L WPN + SHAM group at 6 h (*P* < 0.05) (Table 1), while none of the other groups showed a significant difference. The 0.1 mg/L WPN + SHAM group, 0.1 mg/L WPN group, and 0.08 mg/L WPN + SHAM group showed significantly higher mortalities than the H_2_O group at 12 h (*P* < 0.05) (Table 1). Furthermore, the 0.04–0.1 mg/L WPN + SHAM and 0.08–0.1 mg/L WPN groups showed significantly higher mortalities than that of the H_2_O group at 24 h (*P* < 0.05). All the groups showed higher mortality rates than the H_2_O group at 48 h (*P* < 0.05), except the 0.04 mg/L WPN group (Table 1). The mortality rates in the 0.04–0.1 mg/L WPN + SHAM groups were further compared with that in the 0.1 mg/L WPN group for each time point. No difference was observed in mortality between the 0.04 and 0.08 mg/L WPN + SHAM groups and the 0.1 WPN group at 6 h (*P* > 0.05), while the 0.1 mg/L WPN + SHAM group showed a significantly higher mortality rate than the 0.1 mg/L WPN group (*P* < 0.05) (Table 1; Fig. 7). At 12 h post-treatment, the 0.08–0.1 mg/L WPN + SHAM group and 0.1 mg/L WPN groups showed similar mortality rates (*P* > 0.05) (Table 1; Fig. 7). At 24 h post-treatment, the mortality rates in the 0.06–0.1 mg/L WPN + SHAM groups were significantly higher than that in the 0.1 mg/L WPN group (*P* < 0.05), with 56.31%, 66.40%, and 76.12% increases, respectively (Table 1; Fig. 7). At 48 h post-treatment, the mortality rates of the 0.08–0.1 mg/L WPN + SHAM groups were 100%, and significantly higher than that of 0.1 mg/L WPN group (*P* < 0.05).

In summary, 0.04 mg/L WPN had approximately the same molluscicidal effect as 0.1 WPN at 48 h. At 12–48 h, the molluscicidal effects of 0.06 and 0.08 mg/L WPN + SHAM were higher than that of 0.1 mg/L WPN; and at all time points, the molluscicidal effect of 0.1 mg/L WPN + SHAM was significantly higher than that of 0.1 mg/L WPN.

## Discussion

To the best of our knowledge, this is the first study in which the full-length cDNAs of mollusc *AOX*s have been amplified and confirmed experimentally. The protein sequences of the four AOXs—OhAOX, BgAOX, BaAOX, and BsAOX—were compared with those of 65 AOXs from 63 different species, and the secondary and tertiary structures of the four AOX proteins were predicted. The crystal structure of TbAOX indicates that the active site of AOX consists of a diiron core, four fully conserved glutamic acid residues, and two histidine residues [34]. Mutation of these six amino acid residues could seriously affect the activity of AOX [47]. In this study, the AOXs of all four species of snail contained the same six conserved diiron motifs as the AOX proteins of the other species, which strongly implied that AOX proteins are similar across different species, and correlation between various functions of these proteins.

Earlier studies suggested that AOX is limited to plants, some fungi, and protists; however, more recent studies confirmed that AOXs also exist in the animal kingdom, although they are not found in vertebrates [48]. The phylogenetic tree constructed in this study revealed an evolutionary relationship between the AOXs of different species. The AOX of land plants (monocots and eudicots) and the Euglenozoa formed a separate monophyletic clade with very high bootstrap values, which indicated that the AOXs of these species may have evolved from a homologous AOX carried by their common ancestors in each kingdom. This is consistent with the results of a phylogenetic analysis of AOXs from land plants and the phylum Euglenozoa carried out by Pennisi et al. [49]. Other kingdoms showed certain cross-relationships: metazoans in this study clustered into a large clade, except *L. anatina* in the phylum Brachiopoda. The AOX of *L. anatina* was close to the AOX of the protist phylum Ciliophora and also to the AOXs of the Chlorophyta in the Plantae. AOX has been found in various metazoan phyla [48], and evolved with the rapidly changing concentration of oxygen, hypoxia, and increased H_2_S concentration in the early atmosphere of the Earth [50–52]. AOX is not sensitive to sulphide, and can oxidise panthenol and reduce O_2_ to water when sulphide inhibits CCO, thus maintaining electron transport. When an organism is challenged with environmental stress, the inhibition of the Cyt c pathway often leads to the overproduction of ROS and dysfunction of the mitochondrial electron transport chain. At the same time, AOX can reduce the production of ROS by preventing overpotential of the mitochondrial membrane, thus alleviating oxidative stress [23, 30, 51, 53, 54]. Studies on some bivalve AOXs have shown that AOX transcripts and proteins can be up-regulated and further coordinated with the mitochondrial electron transport chain to support the survival of bivalves under stress conditions [30, 51]. In the present study, OhAOX was close to AOX of *P. canaliculata*, and the AOXs of the three species of *Biomphalaria* were close to the AOX of *A. californica*. Furthermore, all of these AOXs clustered together to form the Gastropoda group, which grouped together with the bivalve AOXs into the bigger clade of Mollusca, with high support values. This suggested that the AOXs of *Oncomelania* and *Biomphalaria* snails, which are in the Gastropoda, may have similar functions to bivalve AOXs in the mitochondrial respiratory chain.

Under WPN stress, the expression of *OhAOX* mRNA in the whole snail and the head-foot region showed a significant increase, and reached its highest levels at 24 h and 12 h. Moreover, western blot analysis revealed a significant increase of OhAOX protein in the whole snail after WPN treatment, although the highest level was observed at 48 h post-treatment. This hysteresis was explained well by the mathematical model used in our previous work [17]. Previous studies showed that the activities of the enzymes CCO and SDH in the Cyt c respiratory pathway decreased during periods of OhAOX increase [14, 55]. These results suggested that the up-regulated expression of *OhAOX* may be involved in regulating oxidative stress in snails under WPN stress, by responding to the inhibition of aerobic respiration caused by a decrease in CCO activity. During this response, AOX can replace CCO to play a role in electron transfer in oxidative phosphorylation, by scavenging electrons and free radicals that accumulate due to reduced CCO activity, and thus protect *Oncomelania* snails from oxidative damage [14, 56]. Although the expression of transcripts of several enzymes that play a role in oxidative phosphorylation in *O. hupensis*, such as NADH dehydrogenase, SDH, CCO, and ATPase, did not change after WPN treatment, that of CCR decreased significantly at 24 h post-treatment, which suggested the inhibition of aerobic respiration. The disagreement between the levels of enzyme transcripts in this study and those of enzymes such as NADH dehydrogenase subunit 3, CCO subunit 2 (COX2), and Na/K transporter ATPase subunit α in previous work on *O. hupensis* [14] could be explained by the variety of different subunits in each MRC. It also indicates that a comprehensive understanding of all MRC subunits in *O. hupensis* is necessary for further investigation of this species under WPN stress.

Tissue localisation showed that *OhAOX* mRNA significantly increased in the muscle of the head-foot after WPN treatment, to a level much higher than that in the other tissues, with a tendency to show first an increase then a decrease, as also shown by qPCR. This further confirmed that the response of *OhAOX* differed in different tissues of *O. hupensis* under WPN stress, and that the muscle of the head-foot was one of the critical effector tissues for the response of *OhAOX* to niclosamide. This tissue-specific response was also observed in other molluscs under stress. The up-regulation of *AOX* in the gills was much higher than that in the digestive glands when *Crassostrea gigas* was re-oxygenated after hypoxia [51], which suggested that *AOX* in the gills may be the first sensor in response to oxygen fluctuations [51]. Similarly, the transcript level of *AOX* in the gills of *Diplodon chilensis* increased fourfold after hypoxia treatment, but no significant difference was found in the mantle and head-foot [30]. In the present study, *OhAOX* in the head-foot region was up-regulated significantly more than in the liver-gonad. This may be related to the fact that the head-foot comes into contact with niclosamide first, as the liver-gonad and other organs are coiled in the shell. WPN may also enter an *Oncomelania* snail through the gill located in the gill pouch on the left of the snail’s neck, and the gill may act as a sensor similar to those in *C. gigas*. Although the increase of *OhAOX* in the liver-gonad was not significant after WPN treatment, it was significantly higher in the liver-gonads of WPN-treated snails than in the H_2_O-treated snails at 24 h. Also, at 48 h after treatment, the expression of OhAOX protein showed a significant increase in the liver-gonad, but not in the head-foot. These results implied that the effect of OhAOX in the liver-gonad is different from that in the head-foot. In addition, this inconsistency between the tissue expression of *OhAOX* mRNA and that of OhAOX protein may be related to the time periods at which they were measured and hysteresis.

The process of normal mitochondrial respiration is the primary source of ROS. ROS include H_2_O_2_, hydroxyl radicals, and superoxide anions, which affect cellular redox homeostasis [27, 57]. When the production rate of ROS exceeds the decomposition rate, oxidative stress occurs, resulting in changes in cell metabolism and oxidative damage to the cell structure [57, 58]. Xiong et al. [14] found that the superoxide dismutase (SOD) activity of *O. hupensis* was significantly higher at 3 h and 12 h after WPN treatment than after H_2_O treatment. This indicated that there was oxidative stress in *Oncomelania* snails after WPN treatment. In the present study, the dynamic change in ROS levels in snails after WPN treatment was estimated using the fluorescence probe DCFH-DA method. The level of ROS in the liver-gonad of snails declined significantly at 6 h after WPN treatment, reached its lowest value at 12 h, and then returned to its initial value. It is currently believed that AOX can reduce the production of ROS by preventing the excessive reduction of ubiquinone in the respiratory chain [59]. H_2_O_2_ production in *Acanthamoeba castellanii* was significantly reduced when purine nucleotides activated the mitochondrial AOX respiratory pathway. However, this effect was weakened when AOX activity was inhibited [28]. Therefore, it suggested that the decrease of ROS content in the liver-gonad of *O. hupensis* may have been related to AOX activation. In addition, cells in the digestive gland of snails contain a variety of antioxidant enzymes, such as SOD, catalase, and glutathione peroxidase, which decompose superoxide anion free radical and H_2_O_2_ [57]. Thus, the reduction of ROS in the liver-gonad suggests that certain antioxidant enzymes in *O. hupensis* were induced to resist oxidative stress in the early stages of responses to niclosamide, which is supported by the increase of SOD enzyme activity seen in snails in Xiong et al. [14]. Yan et al. [58] found that ROS rose to its highest level when *Anadara subcrenata* was exposed to the oxidative stress of Cd and phenylalanine for 5 days, and that it decreased to its normal level at 9 days when the antioxidant enzyme activity and ROS level reached equilibrium. Thus, the fact that the ROS level of the liver-gonad of *O. hupensis* showed no significant difference from 24 to 48 h after niclosamide treatment implies that ROS and the antioxidant system in the liver-gonad of *O. hupensis* might have reached equilibrium in the late stage of the treatment. In addition, no significant change in the ROS level was shown in the whole soft body and head-foot after WPN treatment. This may be explained by limited DCFH-DA, which mainly reacts with specific ROS like hydrogen peroxide and hydroxyl radicals. Other types of ROS, like superoxide, hypochlorite, and singlet oxygen, need to be further investigated in this context.

In the molluscicidal assay, the molluscicidal effect of niclosamide was significantly enhanced when the activity of AOX was inhibited by SHAM, and the combination of WPN and SHAM also benefited an earlier onset of the molluscicidal efficacy of WPN. The earlier AOX was inhibited, the more significant the molluscicidal effect of WPN. In addition, when AOX was inhibited by SHAM, enhanced molluscicidal efficacy was seen simultaneously with a reduced niclosamide dosage. Snails in the 0.08–0.1 mg/L WPN + SHAM groups showed higher mortality than those in the 0.1 WPN group at all time points, and snails in the 0.04–0.06 mg/L WPN + SHAM groups showed close to, or higher, mortality than those in the 0.1 mg/L WPN group after 24 h. More specifically, snail mortality in the 0.06, 0.08, and 0.1 mg/LWPN + SHAM groups increased by 56.31%, 66.40%, and 76.12% at 24 h, respectively, compared to that when 0.1 mg/L WPN was applied alone. This suggested that the mortality of *O. hupensis* could be strongly enhanced by inhibiting OhAOX activity, even when reducing the dosage of niclosamide by 50%.

At 48 h after WPN treatment, up-regulated *OhAOX* had decreased to its initial level. Meanwhile, the mortality rate of *O. hupensis* snails significantly increased to over 90%. This phenomenon was also reported in *Crassostrea virginica*, where up-regulated *AOX* decreased when the oysters were exposed to air for 8 days and faced death [29]. Our results confirmed that the response of *OhAOX* was no longer sufficient to protect *Oncomelania* snails when they faced a very high level of WPN stress, and implied that the most effective time point for AOX inhibition should be further examined.

In addition, the ovarian or testicular stroma of *O. hupensis* showed positive signals of *OhAOX* mRNA and OhAOX protein, as detected by histological localisation. AOX can alleviate developmental abnormalities in *Drosophila* by GeneSwitch, and is involved in cell apoptosis, differentiation, and development [60]. Work in our laboratory showed that the AOX of *B. glabrata* also plays an important role in reproductive development and egg production (data not shown). These results imply that OhAOX expression in the gonad might be involved in its early growth and development and in cell differentiation, all of which needs further investigation.

## Conclusions

The *AOX* genes of *O. hupensis* and three species of *Biomphalaria* were identified in the current study. The typical molecular characteristics and phylogenetic relationships of the AOXs of these molluscs indicated that snail AOXs play a role in the mitochondrial respiratory chain. *OhAOX* mRNA and OhAOX protein significantly increased after WPN treatment, which weakened the molluscicidal effect of niclosamide and counterbalanced the oxidative stress in the snails. The main effect of OhAOX was in the head-foot tissues of snails. AOX in the liver-gonad played a role that may be different from that of AOX in the head-foot. Once the activity of OhAOX had been inhibited, the molluscicidal effect of niclosamide could be significantly enhanced even at lower concentrations of WPN. This study provides information for the development of an environmentally safe snail control method that targets AOX activity. The activity of the AOX pathway in plants under stress, or in thermogenic plants, has been shown to be mainly regulated at the post-translational level [61, 62]. In the present study, post-translation regulation was also implied for natural OhAOX, which was smaller than the recombinant OhAOX. In sum, it is necessary to analyse the activity of AOX in *O. hupensis* with a focus on the mechanism through which it is regulated and identification of the sites that affect it; there is already some indication of the latter from the protein structure deduced in this study.

## Supplementary Information

**Additional file 1: Table S1.** The gene-specific primers used in the 5'- and 3-end RACE-PCR and specific primers used to amplify *OhAOX*, *BgAOX*, *BaAOX*, and *BsAOX*.

**Additional file 2: Table S2.** The species from which the AOX protein sequences were used for the multiple sequence alignments and phylogenetic analysis.

**Additional file 3: Table S3.** Gene-specific primers used in the qPCR for *OhAOX* and other genes related to the mitochondrial respiratory complex (MRC).

**Additional file 4: Figure S1.** The conserved and characteristic domains of OhAOX, BgAOX, BaAOX, and BsAOX with SMART searching.

**Additional file 5: Figure S2.** Secondary structure of the protein deduced from multiple AOX protein sequence alignments with ESPrit. The four snail AOXs in this study are highlighted in yellow on the left.* α* α-Helix,* η* 310-helix,* hash symbol* diiron binding motif. White characters on the red background indicate the complete identical protein sequences, blue frames indicate the homologous regions; the characters highlighted in blue represented the specific C-terminal motifs (NP-[YF]-XPG-[KQE]) in animal AOXs.

**Additional file 6: Figure S3.** The diiron core in the deduced tertiary structure of OhAOX, BgAOX, BaAOX, and BsAOX proteins. Each protein’s monomers are shown as chains 1 and 2. The diiron centre with two iron atoms and an -OH is coordinated with four glutamates (E) and two histidines (H).

**Additional file 7: Figure S4.** Expression and identification of recombinant OhAOX protein in SDS-PAGE and the specificity of anti-OhAOX polyclonal antiserum in western blot. **a** Expression of recombinant proteins.* M* protein marker,* lane 1* uninduced *Escherichia coli* with recombinant OhAOX–pGEX-4 T-1,* lane 2* induced *E. coli* with pGEX-4 T-1,* lane 3* precipitate of induced *E. coli* lysis with recombinant OhAOX–pGEX-4 T-1,* lane 4* supernatant of induced *E. coli* lysis with recombinant OhAOX–pGEX-4T-1,* lane 5* induced *E. coli* with OhAOX–pGEX-4T-1. **b** Purification of recombinant protein.* Lane 1* Supernatant of induced *E. coli* lysis with recombinant OhAOX–pGEX-4 T-1,* lane 2* flow-through after GST beads binding,* lanes 3–5* washing solution,* lanes* 6–8 elution of recombinant protein. **c** Total protein of snail and purified recombinant OhAOX.* Lane 1* Total protein extracted from whole *O. hupensis* snail,* lane 2* purified recombinant OhAOX without GST tag. **d** Specificity of mouse anti-OhAOX serum by western blot.* Lane 1* Total protein of *O. hupensis* snail,* lane 2* PBS,* lane 3* purified recombinant OhAOX protein,* lane 1'* control for lane reacted with mouse PBS immune serum,* lane 3'* control for lane 3 reacted with mouse PBS immune serum.

**Additional file 8: Figure S5.** Tissue distribution of *OhAOX* mRNA in untreated *O. hupensis* snail by in situ hybridisation**.** Upper image is from the whole snail, and the lower images are from the head-foot region (left) and liver-gonad. The stained purple-blue area was identified as positive for *OhAOX* mRNA.* G* Ganglia,* HF* head-foot,* L* liver,* M* muscle,* P* pellicle,* T* testis.

**Additional file 9: Table S4.** Mortality rate (%) of snails after WPN treatment, with the AOX inhibitor (SHAM) added at 0 h or 6 h.

## Data Availability

The sequences of the four AOX genes identified in this study have been deposited in GenBank under accession numbers MZ436164-MZ436167. Most of the data generated or analysed during this study are included in this published article and its supporting files. Any related data are available from the corresponding author on reasonable request.

## Abbreviations

AOX: Alternative oxidase
ATP: Adenosine triphosphate
BaAOX: *Biomphalaria alexandrina* alternative oxidase
BgAOX: *Biomphalaria glabrata* alternative oxidase
BsAOX: *Biomphalaria straminea* alternative oxidase
CCO: Cytochrome c oxidase
CCR: Cytochrome c reductase
Cyt c: Cytochrome c
DCF: Dichlorofluorescein
DCFH-DA: 2′,7′-Dichlorodihydrofluorescein diacetate
HRP: Horseradish peroxidase
IOD: Integrated optical density
MRC: Mitochondrial respiratory complex
NADH: Nicotinamide adenine dinucleotide
OhAOX: *Oncomelania hupensis* alternative oxidase
PBS: Phosphate-buffered saline
PCR: Polymerase chain reaction
qPCR: Quantitative real-time polymerase chain reaction
ROS: Reactive oxygen species
SDH: Succinic dehydrogenase
SDS-PAGE: Sodium dodecyl sulfate–polyacrylamide gel electrophoresis
SHAM: Salicylhydroxamic acid
SMART: Simple Modular Architecture Research Tool
SOD: Superoxide dismutase
TbAOX: *Trypanosoma brucei* alternative oxidase
WPN: Wettable powder of niclosamide
