# Supplementary figures and images for: The identification of alternative oxidase in intermediate host snails of Schistosoma and its potential role in protecting Oncomelania hupensis against niclosamide-induced stress
Source: Parasit Vectors. 2022 Mar 21;15:97. doi: 10.1186/s13071-022-05227-5 (PMC8935807; doi:10.1186/s13071-022-05227-5)

OhAOX

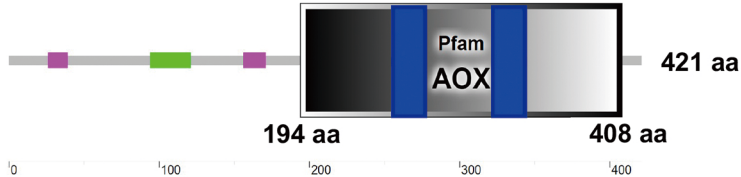

BgAOX

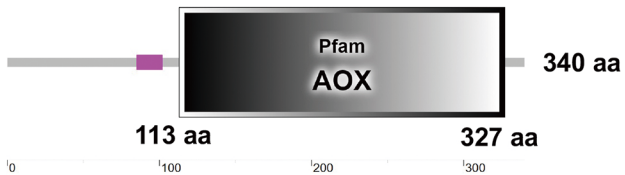

BaAOX  
BsAOX

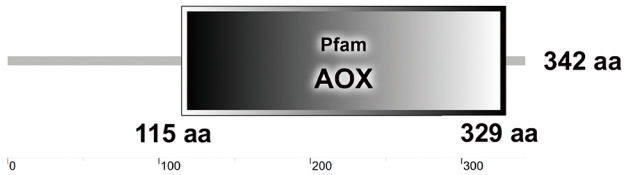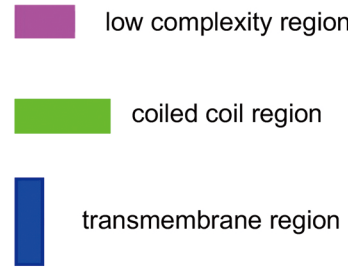

Supplement: Supplementary file 4 — Additional file 4: Figure S1. The conserved and characteristic domains of OhAOX, BgAOX, BaAOX, and BsAOX with SMART searching. [file 13071_2022_5227_MOESM4_ESM.pdf]

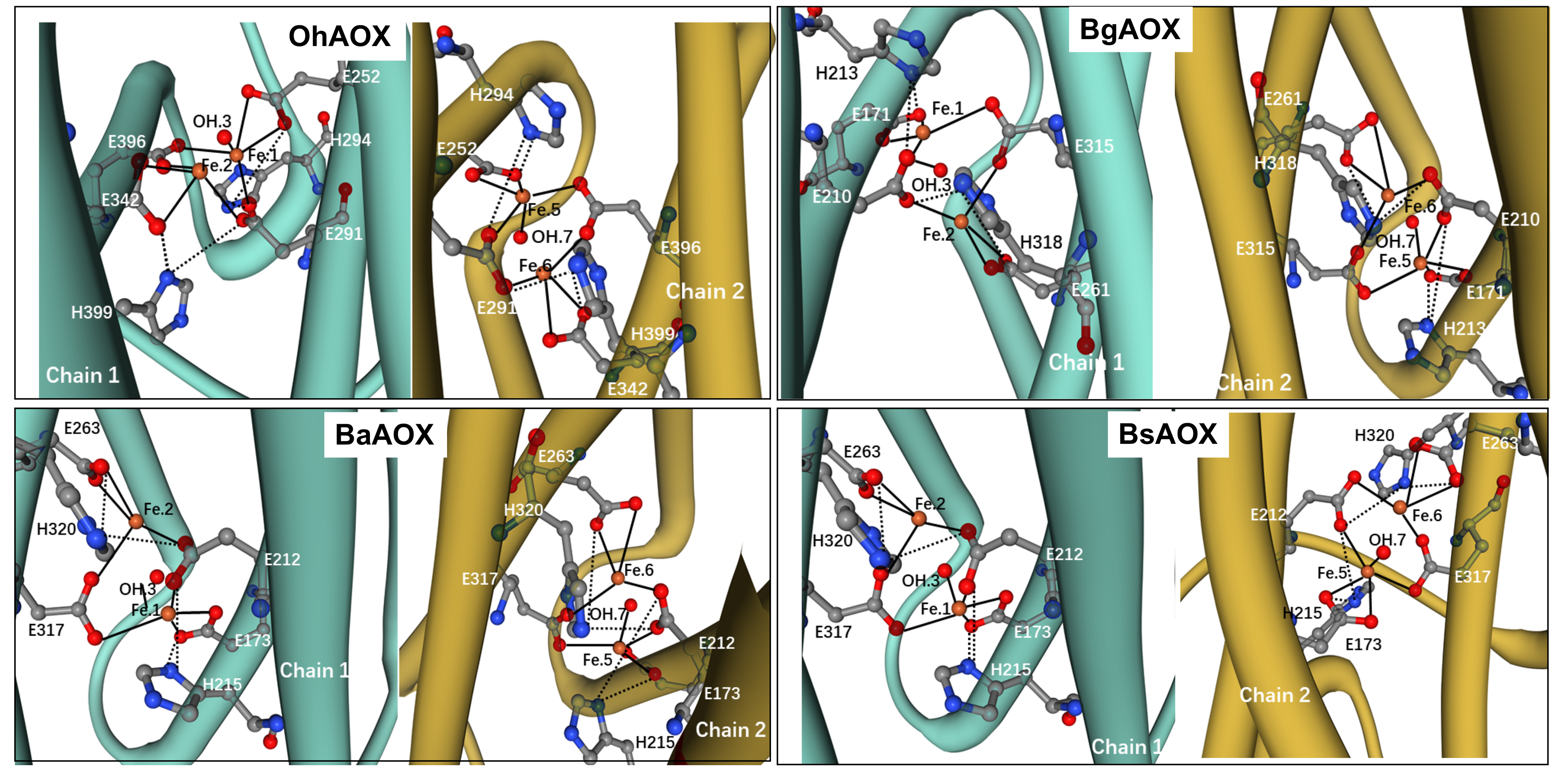

Supplement: Supplementary file 6 — Additional file 6: Figure S3. The diiron core in the deduced tertiary structure of OhAOX, BgAOX, BaAOX, and BsAOX proteins. Each protein’s monomers are shown as chains 1 and 2. The diiron centre with two iron atoms and an -OH is coordinated with four glutamates (E) and two histidines (H). [file 13071_2022_5227_MOESM6_ESM.tif]

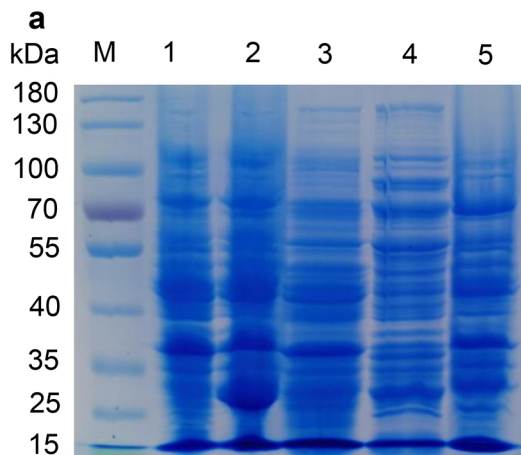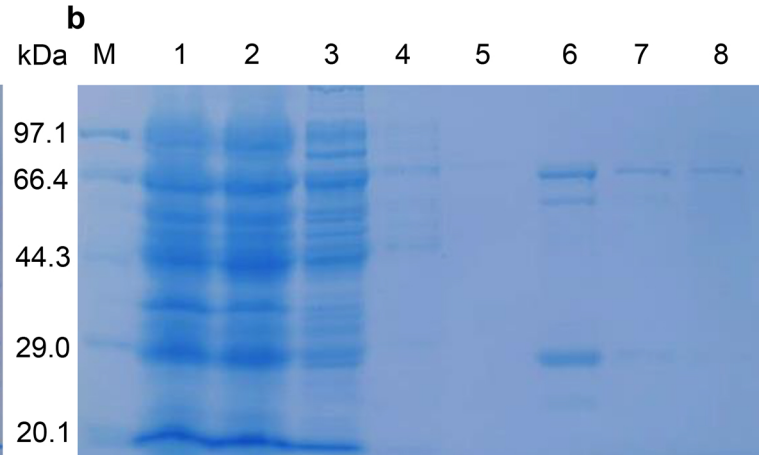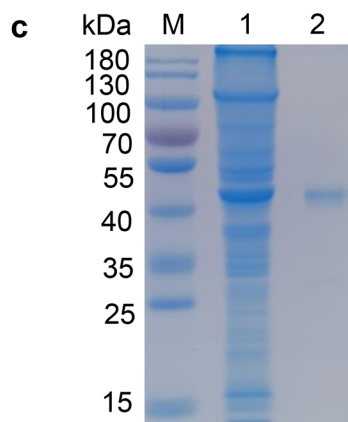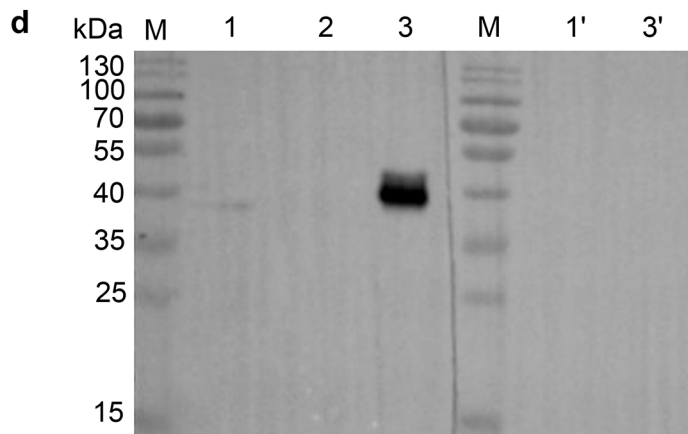

Supplement: Supplementary file 7 — Additional file 7: Figure S4. Expression and identification of recombinant OhAOX protein in SDS-PAGE and the specificity of anti-OhAOX polyclonal antiserum in western blot. a Expression of recombinant proteins. M protein marker, lane 1 uninduced Escherichia coli with recombinant OhAOX–pGEX-4 T-1, lane 2 induced E. coli with pGEX-4 T-1, lane 3 precipitate of induced E. coli lysis with recombinant OhAOX–pGEX-4 T-1, lane 4 supernatant of induced E. coli lysis with recombinant OhAOX–pGEX-4T-1, lane 5 induced E. coli with OhAOX–pGEX-4T-1. b Purification of recombinant protein. Lane 1 Supernatant of induced E. coli lysis with recombinant OhAOX–pGEX-4 T-1, lane 2 flow-through after GST beads binding, lanes 3–5 washing solution, lanes 6–8 elution of recombinant protein. c Total protein of snail and purified recombinant OhAOX. Lane 1 Total protein extracted from whole O. hupensis snail, lane 2 purified recombinant OhAOX without GST tag. d Specificity of mouse anti-OhAOX serum by western blot. Lane 1 Total protein of O. hupensis snail, lane 2 PBS, lane 3 purified recombinant OhAOX protein, lane 1' control for lane reacted with mouse PBS immune serum, lane 3' control for lane 3 reacted with mouse PBS immune serum. [file 13071_2022_5227_MOESM7_ESM.pdf]

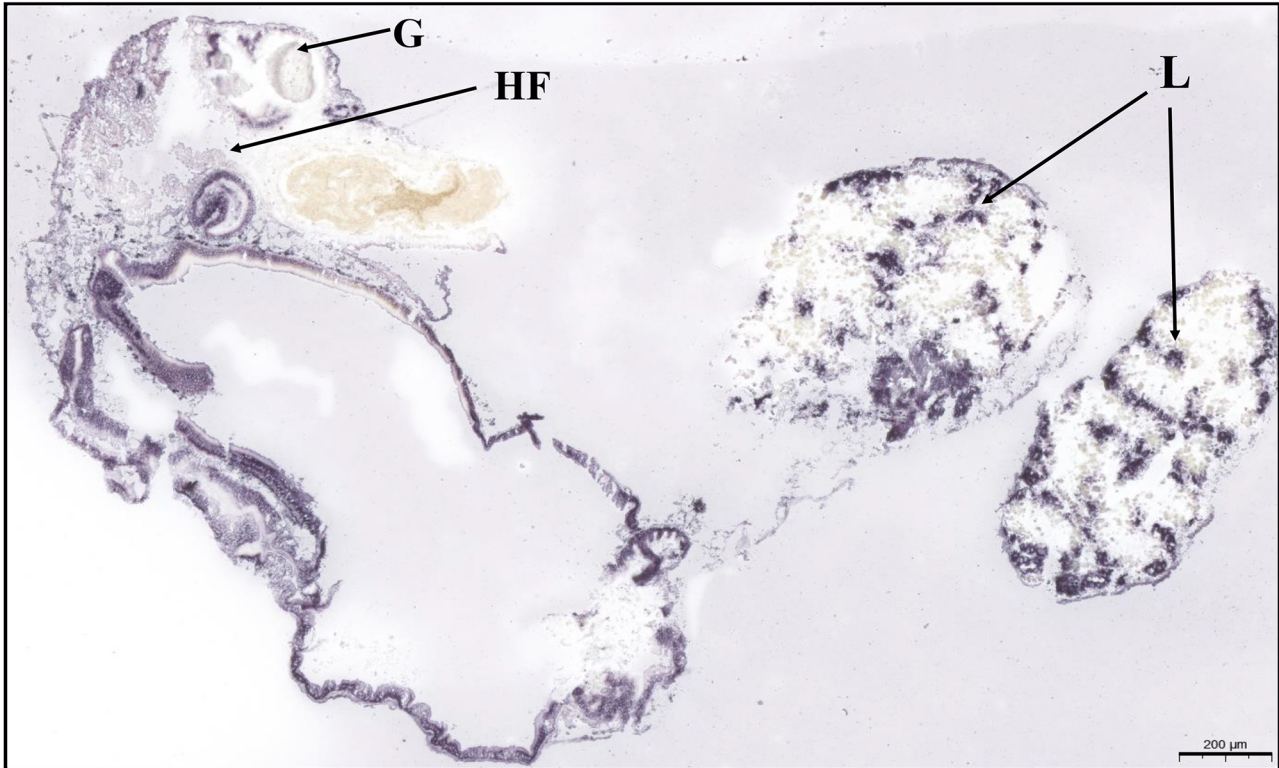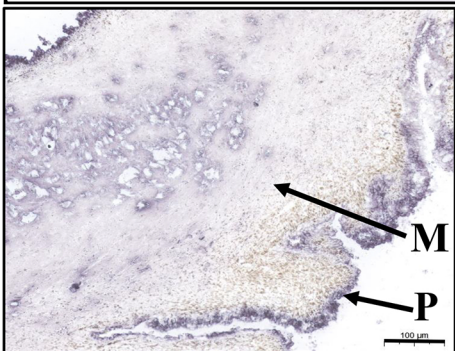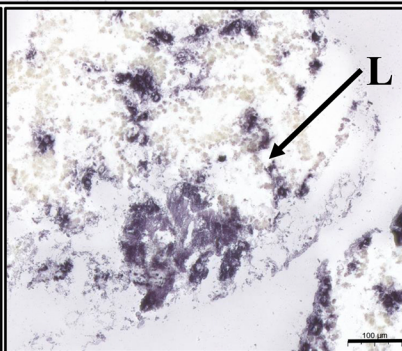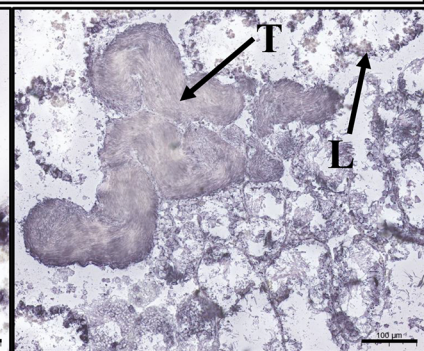

Supplement: Supplementary file 8 — Additional file 8: Figure S5. Tissue distribution of OhAOX mRNA in untreated O. hupensis snail by in situ hybridisation. Upper image is from the whole snail, and the lower images are from the head-foot region (left) and liver-gonad. The stained purple-blue area was identified as positive for OhAOX mRNA. G Ganglia, HF head-foot, L liver, M muscle, P pellicle, T testis. [file 13071_2022_5227_MOESM8_ESM.pdf]
