# Supplementary material for: The identification of alternative oxidase in intermediate host snails of Schistosoma and its potential role in protecting Oncomelania hupensis against niclosamide-induced stress
Source: Parasit Vectors. 2022 Mar 21;15:97. doi: 10.1186/s13071-022-05227-5 (PMC8935807; doi:10.1186/s13071-022-05227-5)
Supplement: Supplementary file 5 — Additional file 5: Figure S2. Secondary structure of the protein deduced from multiple AOX protein sequence alignments with ESPrit. The four snail AOXs in this study are highlighted in yellow on the left. α α-Helix, η 310-helix, hash symbol diiron binding motif. White characters on the red background indicate the complete identical protein sequences, blue frames indicate the homologous regions; the characters highlighted in blue represented the specific C-terminal motifs (NP-[YF]-XPG-[KQE]) in animal AOXs. [file 13071_2022_5227_MOESM5_ESM.pdf]

T. brucei

Species	Sequence (positions: 1 … 10 marked above *T. brucei* M and I)
T. brucei	....MFRNHASRITAAAAPWV
T. congolense	....MLRINASCINAAAATTT
T. vivax	....MLRYRTPTLAAAAAMKV
A. deanei	....MFPLRRQVTARLTTGFTQRGFGATT
C. andersoni	....MNFQSNISRNIISNFNQLFFNCNYIKAT
C. tyzzeri	....MYYVRNLLNTNKLRYFYGRHLWLLSSKVNLI
B. alexandrina	....MNVVSIIRGLTKFGSSNSLVASLLPSTSTFT
B. straminea	....MNVVSIIRGLTKFGSSNSLVASLLPSTSTFT
B. glabrata	....MNRVSIIRGFTKFGSSNSIASLLPSTFN..T
A. californica	.............................MMLSLPSTMRAFAAHGSNFSPMATAFVTSTTGRSFSSSVSGTVRSSSKTLP
P. canaliculate	....MSSSTIFLRSSARTLCASRIVISAAELNLSSAP
O. hupensis	....MSLCFSALTIRPSACAVGALRSSFGASALKLSAHL
P. maximus	....MLTSRMLVRCGAENALKNITAIAPP
M. yessoensis	....MLTSRMLVRCGAENALKNVTIIAPS
C. gigas X2	....MGSLRQITKLSENGVR
C. gigas X1	....MGSLRQITKLSENGVR
C. virginica X2	....MGSLRQITRLSENGVR
C. virginica X1	....MGSLRQITRLSENGVR
B. floridae	....MATRGALRLRTAAVGGMLSRRSFHLSAAPLNSCLLGPRERQ
A. planci	MLTVTAKQASLCANN.LIHVWPRSFSVAVRALND.SITGRPLGLRDPSPCQAAGACRPYSLITGGLSRPCTAKLTSTRTA
P. miniata	MLKVTVKQASLLSANNLVYVWPRCFGLAVHSSSD.SFPSAPR.RPKVSPRLAAVPCRPFSLIAGGSSGSCTAGFALTRAV
A. rubens	MLPLTSKSLLPSSITRVIHVGAVNLSCESGRLATRKFLCVISTGSHHTPSVVSFHGRASPLNHGSYCDPGKTITAGFGCS
A. japonica X1	....MVASSMLYKGSSCFARMSMIGLSPA
S. pistillata	....MATIGLQRVPFSVRGIANCSTFVKP
O. faveolata	....MATIGLRRISVSIRGIINCSSFVKH
A. tenebrosa	....MIVPKLGRFHSSGSFAGSRSVLAII
E. diaphana	....MTTIRIGRVYGSGRLLNAYVALAKQ
N. vectensis	....MSVREFHRAIPLSRILNRSTRSCPT
A. millepora	....MALLKSHRLLSPVVNVVRSAGSTR.
P. damicornis	....MLPTKTRQFFSPTILFALKSSLNRR
D. gigantea	....MKSVSRSLSFFRQHGFRKASSRFITATGKRRWDLP
L. anatina	....MAARGFRQLAGTCRTGCLHCRLRLV
P. dicentrarchi	....MQSFARKFCTSSALVHHSNNALF
A. udagawae	....MNSITATMPLRATALPKT
P. citri	....
H. capsulatum	....MYPTSAGCARVLMACPAPAML
E. crescens	....MYPTSAGSTRILMACSAPSML
P. hystricis	....MSGTTPSICIYKSLLRGA
T. interdigitale H6	....MHAPTPAPFRALVQGSGARLL
E. mesophile	....MNPSRATLLPSL
A. bisporus	....MRYVGTVSTLSYLPRR
P. conissans	....
C. cinerea	....MLKSTLLRTTAVQLRTPTTLSTARLLA
G. lividus	....MTTLLRSALAHSPARHHSLIFRSLLIDHGAYRSTPELRVL
P. ammoniavirescens	....MSVLLRSTLIHSATRHHPLIFRSALIGCGAYRRTPELRVA
S. rosetta	....MTVLLRASRSLLLQQVLLRRP
C. cylindracea	....MLSTSRRFASFYGYRFGAVVSEQLTHG
H. sp. ATCC 50920	....MLRASLTSVSGLLARNAASVSIIRNEFVTASTTALR
O. tauri	.................................MMRRVAQTLARSHLHASTTSQTTTARALWSSGMTRTNFNSTIATTHR
I. sp. A28L	....
A. shirensis	....
M. indica	....
C. papaya	....MNRFVARSVIRALMVNSRSCGGSNRMLYAATTATVR
C. olitorius	....MNRFVVRSVMQGLINGRSSIINGQVCRAAVVGRGVQ
G. max	....MKLTALNSTVRRALLNGRNQNGNRLGSAALMPYAA
V. unguiculata	....MKFIALSCTVRRALLNGRNCNG..LGSTAVMAYAA
M. sativa	....MKHSALCYMARRALIGGRNSNRQSSAVVRSFAAAE
D. carota	....MNQMVARSVIRRLINSQKSPISTFRSHDDIA
S. lycopersicum	....MNQLLVRLVMRRLVSGGTRNGCISSSSAAVMFREVHKPS
A. thaliana	......................................MSQLITKAALRVLLVCGRGNCNMFVSSVSSTSVMKSPYEITA
A. tauschii subsp. strangulata	....MSSRMAGSVLLRRAGAGASRLFATTPSPAA
T. aestivum	....MSSRMAGSVLLRRAGAGAGRLFATTASPAA
H. vulgare subsp. spontaneum	....MSSRMAGSAILRRAGVSAGSLFATTVSPAA
P. hallii	....MSSRAAGSVLLHHLGPRVFGPVSSPAAAAP
S. officinarum	....
P. dactylifera	....MMSSRMAGSALIRQLGPRLFSAG...ATRAA
E. guineensis	....MMSSRMTGSALLRQLGPRLFSVSSAAPTRAA
P. equestris	....MMSSQVRRSVTLVRQLGLRSFSTSPSADRVGA
M. alkaliphilum	....

T. brucei

	20
T. brucei	LRTACRQKSDA..K
T. congolense	LWSAMRLRSDG..K
T. vivax	QQMFTHSSSSSN...K
A. deanei	LRCCAATQESAI...P
C. andersoni	NKCITTSRYVSTIDTRVGQTSVYSPLHQP...SQPHPD
C. tyzzeri	NLCSIVHSNKGQKITSKLYITLEKDRSNN...NQGDFS
B. alexandrina	YKNGKKLLCQRTALRSSVIGSYQFLVMQN......................................RGIADDKQ.....
B. straminea	YKNGKKLLCQRTALRSSVIGSYQFLVMQN......................................RGIADDKQ.....
B. glabrata	HKNGKKLLCQRTGFRTSFIEGYQFVVVQI......................................RGIADDKQ.....
A. californica	IRHASCVMCTTDQAKPAHKVTSSFVGLQQ......................................YRGLADKKEINEK
P. canaliculate	AVGR........LVCAVAPQVRHLTTQR...EIREN
O. hupensis	SSCARTVDVGAVGASIQFPQIRTLSTDGRITERTFASKPKHTKCADCTCATCSNKVRMLSTTETQLQQVEGNLKKQIQEN
P. maximus	VKVKQGFVVN.HLWLHRAPVRPDMCSVRN..YVATKEEVDDH
M. yessoensis	QSVKVKQGCVGHLHLAPARPTLCSATVRN..YAATKQEVDEH
C. gigas X2	IFCSQLKNLENNSILLRVSGIRTSNGLR...NAGTKADVDEN
C. gigas X1	IFCSQLKNLENNSILLRVSGIRTSNGLR...NAGTKADVDEN
C. virginica X2	IFCKQMKDAENASILLRINGIRTGSGLR...TAATKADDEN.
C. virginica X1	IFCKQMKDAENASILLRINGIRTGSGLR...TAATKADDEN.
B. floridae	ISLVQRMYLSSATQTSPKPVTEKKVEKVT..................................VVSTPAQKDEIQMKDAR
A. planci	GKNILAQPFNLSSSSTLKQRFVSTTSPQ...DET
P. miniata	GSNVVLNPFNVTNPTTHQKIQSLSTASQQ..DEKVRVDMP
A. rubens	STSSAGLKRLALVGCIHIQQAKLLSTSP...P
A. japonica X1	KTSLWLGKGQLTKACRNHKQLLSTSCMAL..QG.......
S. pistillata	ISAVDFFG...KLWGSARPRRTGVS..QF..STNTILKRS
O. faveolata	INVTLAESDSFKLRGILLPQRTAIS..LL..STNAIKR..
A. tenebrosa	LARGP..RLGAVNIRSCCQMQGGYFPKIS..TPSAPYSVR
E. diaphana	QRACFGQTAHLANNIQIQRSHAGKSSGDF..VVSSVPPVR
N. vectensis	RLVLRG..LRSSSFASSLKQRTAFFTANR..LTPAYLTTN
A. millepora	................SGFLLGIRSQESL..RKPTLCRLL
P. damicornis	ILPGFR..........ALQDGGLLLETPL..SNSCTVRLL
D. gigantea	HSTCHINIWPGHRSFSHIVRQQKHSSPAI..ARNKFQKYS
L. anatina	MEMQRITPVVSTIHARQICYTQPRPAVSA...II
P. dicentrarchi	KQSINYTPLY..
A. udagawae	YLRFTIRTYASAVGASGCSRPLLASSN..H
P. citri	MLSRQSATQAIRVITNSTLPLNASRAI..F
H. capsulatum	RGPLLRPSTTAIRGLRGSP..LLYHYA..T
E. crescens	RG.ALRPSTSAMRGLRSSP..MIYHYT..A
P. hystricis	AGGMLRQSTAVGTGLRGSP..VLLHQF..G
T. interdigitale H6	SQKCLASGFRPSTLAIGLRGNSASLYN..Q
E. mesophile	TTNISRVASKGLYCSAATTGLRASYSA..L
A. bisporus	IGFMPAGRRTLLTSSDKETLNSSAVEK..H
P. conissans	....
C. cinerea	RPLVCRGLHSTGYLRSALSASAKTRET..H
G. lividus	VTKRSNIHTGRVDQNFLTKKGPNEKAA..T
P. ammoniavirescens	VIQGSNIHTGRIDQNSLIKKRLNEEAD..T
S. rosetta	CVRVVTASNALAATSQVRGVHTSKDNE..Q
C. cylindracea	PLQNTYFSTQQQPLRKTLDLEWDEDKH..D
H. sp. ATCC 50920	GRLFTTSPTSWSDLPPMAEPPAPHAGL..R
O. tauri	DEVAMDGSARRARAHSRAPGAPLASGR..S
I. sp. A28L	....
A. shirensis	....
M. indica	.....................MLSNAGGAEAQVK......................................EQK.....
C. papaya	PWEISDVGRGGDFGAFNWRRMMSSQESSAVEEVK......................................KEKSAVEQ
C. olitorius	LSGQQNGMPFASVELRRMMSTASAPASMGKVPLA......................................TEEKKENP
G. max	AETRLLCAGGANGWFFYWKRTMVSPAEAKVP..EKEKE
V. unguiculata	PETRFLCAGAANGGLFYWRRSMASQAEAKLP..EKDKE
M. sativa	IGQRHLYADGGNGGFFYWKRMMSSQAAPSKPSAEE.................................TEAKSTEKNEKK
D. carota	IANRQRLGIIGGGARVFGTRMMSAAGE...
S. lycopersicum	SESVVMMSSQRLRFSGRWWRMMCSTTSKNSTKSSD.................................VRKEDEKLSGDS
A. thaliana	PMRIHDWCGGFGDFKIGSKHVQGNFNLRWMGMSSA.................................SAMEKKDENLTV
A. tauschii subsp. strangulata	RAVLG.......GGEGAWVRLMSTSAAS......................................QVKDEAAKAVKAEA
T. aestivum	RTALG.......GGGGAWVRMMSTSAAS......................................QVKDEAAKGVKAEA
H. vulgare subsp. spontaneum	RTDLG.......GGRGAWVRMMSTSAVS......................................PIKDEAAKVDNAEA
P. hallii	RPLLALAGGGERGAAAVWVRLLSTAAAEAKEEAAASKGS..........AGSTVAAKAEAAASKGNAGSTAAAKAEAAEA
S. officinarum	....
P. dactylifera	AAGEPSYTLLLTGSAGPAAAKPPVAALMRLFPAR...............MASTTAAAPADGSEEASEPSKSAAAADAAKE
E. guineensis	AAVEPSYTLLLTGSASPATAKSPVAALFRLFPAR...............MASTTAAKPAVGGEEESEPSK......PAAE
P. equestris	PLNSFLFGSGTSSAAPTAIVRLLQVRMASTSALP...................................EPIGGEDDRSP
M. alkaliphilum	....

T. brucei	α1 η1 η2
	30 40 50 60
**T.** ***brucei***	TPVWGHTQLNRLSFLETVPVVPLRVSDESSE...
**T.** ***congolense***	APVWGHTQLNRLSFIDAVPAVPQRAGDESSE...
**T.** ***vivax***	TPVWGYTQVNRLSFVDLVPRVPAREEDESSS...
**A.** ***deanei***	QHQWGSRQLNQLSFLSFAKRLSTKEHVEIAEN..SY
**C.** ***andersoni***	KSMNGTHYYHHMNNESQQLYSAPHFRKGIQTL...................................KSEDEGEVKEYSF
**C.** ***tyzzeri***	KKRTLECKSDQINKFDAENEEKVGSHFMKKSN...................................HAASILEGKEYGF
**B.** ***alexandrina***	....TESNYGKLPNPS..DLAHFRSSSSPPVQ................................KDDKEKDNPPIGIYAM
**B.** ***straminea***	....TESNYGKLPNPS..DLAHFRSSSSPPVQ................................KDDKEKDNPPIGIYAM
**B.** ***glabrata***	....TETKYDKLPNPS..DLAHFRSSSSPPVK................................KDDKEKDNPPIGMYAM
**A.** ***californica***	VDEYRKGDFDTIPDPG..NLVHFKTKDALEKTP.........................KTDVQDKGKKDKEMPPIGTYVL
**P.** ***canaliculate***	IKDYREGKFDKIPDPG..HLEHFRNKSERKVKVAEEGERSQQGAQQVQGVQNVPGVDGEQSVQGAQGHINEEPPMGVYAM
**O.** ***hupensis***	VKQAEEGKFNTMPDPG..HLEHFMKRSQK....TEVSSVHQSATPKSGISQSSDSRSSVNQSATDISIFESEPPMGVYTL
**P.** ***maximus***	VKKYKEGNFEKIPDPG..HLEHFKTSEALK..............SVS..TTTAATTVTKVTGSTSEVTETYHPPVGTHAL
**M.** ***yessoensis***	VRKYKEGNFDKIPDPE..RLEHFKTHEALK..............TASSATVATAAGKAKVTGSTTDVTNDSPPPVGTHAL
**C.** ***gigas X2***	IKKFKEENFEKIPDPE..QLDHFRK.................................TQSTDQLVESMKNPPPMGTHTL
**C.** ***gigas X1***	IKKFKEENFEKIPDPE..QLDHFRK.................................TQSTDQLVESMKNPPPMGTHTL
**C.** ***virginica X2***	IKKFKEGNFEKIPDPN..HLDHFRK.................................SQSTETLVESLKDPPPMGTHTL
**C.** ***virginica X1***	IKKFKEGNFEKIPDPN..HLDHFRK.................................SQSTETLVESLKDPPPMGTHTL
**B.** ***floridae***	GTTVPLDVLSSVPDHHRPDLAHFRTG...............................LSKPDDLEKKHVKDDGTMGQYSL
**A.** ***planci***	AGDSTKQKPKLAPHFRISSGK...TSAGDIPHPKAT.............................TDEDENGEDSRRYLL
**P.** ***miniata***	AEDDKKEAKKHAPHFRIALKKQPEFSGDEMSHPKAT.............................TDDSKEGEDDRQYLL
**A.** ***rubens***	QGDDKGVHKQEGDVGEIAPAPHFRRTGIEKKAEEEA.............................KSKELEKDEDGQYLL
**A.** ***japonica X1***	VQGKKQTSSGLAPHFKR..............NLDTN.............................KGNQEAATDTVDYLL
**S.** ***pistillata***	ASEVQQSTPAVTTQSKEKPIGHFGKNGSTAG...DENYRM
**O.** ***faveolata***	EPADEQSTPQVTTQFKEIVIDHFGKDGNTTG...NEEYRM
**A.** ***tenebrosa***	TMYTSSYCCQNLPHFKESQNDAACKDSKKE..DSPYLL
**E.** ***diaphana***	SLFTTTTNQQNLPHFKES.LDAKPSSANAD..DRSYLL
**N.** ***vectensis***	LFHLSVSDAQHLPHFKENPQQPTGKSNDPD..DRPYLL
**A.** ***millepora***	SISIVCTEKDSSPHFRERTLANEAEGGDQTLG..NEVYRM
**P.** ***damicornis***	STTGVSRQKD.LKHFKAGAEKKDSVDNDHTRG..NEIYRM
**D.** ***gigantea***	SSSSNVSNQQSTPTHFKELTGKHPLPEVEEEKQIE....................................REKAKDYIL
**L.** ***anatina***	AEAKERSKREDYEHFRMKEYREADEKAGKHTS..GDDYLL
**P.** ***dicentrarchi***	.......QMSVKQHFKVTKKAMAQQEKQTIDS..KKLYSF
**A.** ***udagawae***	FQSITKRPISSTPQTQIKDYFPPPK..APHIK..EVETAW
**P.** ***citri***	VNPSSRRDFSTSKPAMMKEFFPEAD..APHVK..RTQAAW
**H.** ***capsulatum***	TSNNNVRYFSSTSRRWIKEFFAPPK.ETDHIV..ESVTTW
**E.** ***crescens***	SNNNNTRCFSSSPSRPIREFFPPPKNTKHVLV..ESVTTW
**P.** ***hystricis***	ISNN.RRCFSITSPSRIKEYFPIVK..TKHIV..ETETTW
**T.** ***interdigitale*** **H6**	NNHDQGRNFSTTSPARLKEFFAAPK.QTKRIV..ETESAF
**E.** ***mesophile***	RPLASSRHRNFSSSQQTQLDFFPPPKNLPNIK..FTPPSW
**A.** ***bisporus***	AAERRNIASTSPAKETSELTKQSAATTIPTMV..RGDWVL
**P.** ***conissans***	..............................MV..RGDWVL
**C.** ***cinerea***	TETRSTKSETKEAPKADPLTTESAVSTVPTMV..RGDWVL
**G.** ***lividus***	GTEGFHREDLAPSKKALTDSKLDTVNTVPVEV..RGDWVL
**P.** ***ammoniavirescens***	GTEGFHPGDLTPSKKALTEP..DAVSTVPVEV..RGDWVL
**S.** ***rosetta***	LEGKVHDVSVQHFRRSNNPHPLFSKVDDHGNP..VWVNPV
**C.** ***cylindracea***	FAK.SKESTSKTPPLSHPSSQKREHGDT..TYFL
**H.** ***sp.*** **ATCC 50920**	GGS.LGQPSLNEDGSAKVSDAEDAWGGP..GYML
**O.** ***tauri***	FASSAAQPAPKEDKATEVANAKRLDDPPRGAS..QGSYML
**I.** ***sp. A28L***	....
**A.** ***shirensis***	....
**M.** ***indica***	....EEKKDAMVSNYWGIS..RPKITREDGSE..WPWNCF
**C.** ***papaya***	AKKDSSGDKMMLSSYWGIS..RPKITRKDGTE..WPWNCF
**C.** ***olitorius***	ATEEQKAKDMVVSSYWGIS..RPKITREDGTE..WPWNCF
**G.** ***max***	KEKAKAEKSVVESSYWGIS..RPKVVREDGTE..WPWNCF
**V.** ***unguiculata***	K..AEAEKSVVESSYWGIS..RPKIMREDGTE..WPWNCF
**M.** ***sativa***	EESSGTKNNVVVSSYWGIS..RPKIMREDGTE..WPWNCF
**D.** ***carota***	.NEAAKENKVSVTSYWGVA..RPKVKREDGSD..WPWNCF
**S.** ***lycopersicum***	VEKMEGEDEVALSSYWGVS..RPKITKEDGSV..WPWNSF
**A.** ***thaliana***	KKGQNGGGSVAVPSYWGIETAKMKITRKDGSD..WPWNCF
**A.** ***tauschii subsp. strangulata***	AKGDGEKKEVAISSYWGIE.QSKKLVREDGTE..WKWSCF
**T.** ***aestivum***	AKGDGEKKEVAISSYWGID.QSKKLVREDGTE..WKWSCF
**H.** ***vulgare subsp. spontaneum***	AKDEGEKKDVAISSYWGID.QLRKLARDDGTE..WKWSCF
**P.** ***hallii***	VKEGDGKKSPVVNSYWGIE.PS.KLVNKDGVE..WRWSCF
**S.** ***officinarum***	.........................MSKDGVE..WRWSCF
**P.** ***dactylifera***	PSAAPANERKAITSYWGIN.PN.KVVKEDGTE..WRWSCF
**E.** ***guineensis***	SSDASPSERKAIASYWDIN.PN.KIVKEDGTE..WKWSCF
**P.** ***equestris***	KGQANPSGSNQMTSYWGVA.PR.KLFKEDGSE..WRWSCF
**M.** ***alkaliphilum***	....

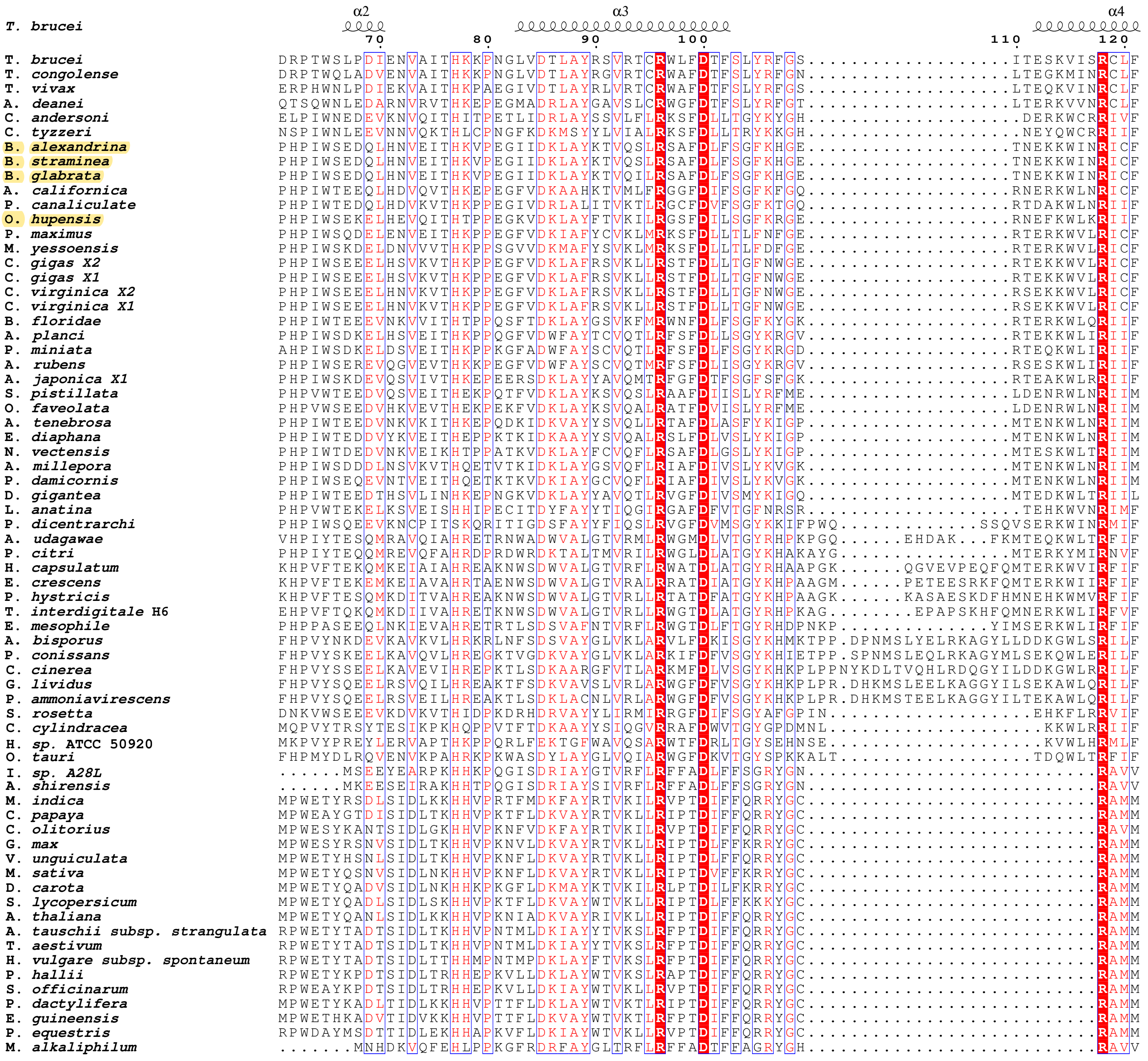
T. brucei
α2
α3
α4
70
80
90
100
110
120
T. brucei
T. congolense
T. vivax
A. deanei
C. andersoni
C. tyzzeri
B. alexandrina
B. straminea
B. glabrata
A. californica
P. canaliculate
O. hupensis
P. maximus
M. yessoensis
C. gigas X2
C. gigas X1
C. virginica X2
C. virginica X1
B. floridae
A. planci
P. miniata
A. rubens
A. japonica X1
S. pistillata
O. faveolata
A. tenebrosa
E. diaphana
N. vectensis
A. millepora
P. damicornis
D. gigantea
L. anatina
P. dicentrarchi
A. udagawae
P. citri
H. capsulatum
E. crescens
P. hystricis
T. interdigitale H6
E. mesophile
A. bisporus
P. conissans
C. cinerea
G. lividus
P. ammoniavirescens
S. rosetta
C. cylindracea
H. sp. ATCC 50920
O. tauri
I. sp. A28L
A. shirensis
M. indica
C. papaya
C. olitorius
G. max
V. unguiculata
M. sativa
D. carota
S. lycopersicum
A. thaliana
A. tauschii subsp. strangulata
T. aestivum
H. vulgare subsp. spontaneum
P. hallii
S. officinarum
P. dactylifera
E. guineensis
P. equestris
M. alkaliphilum

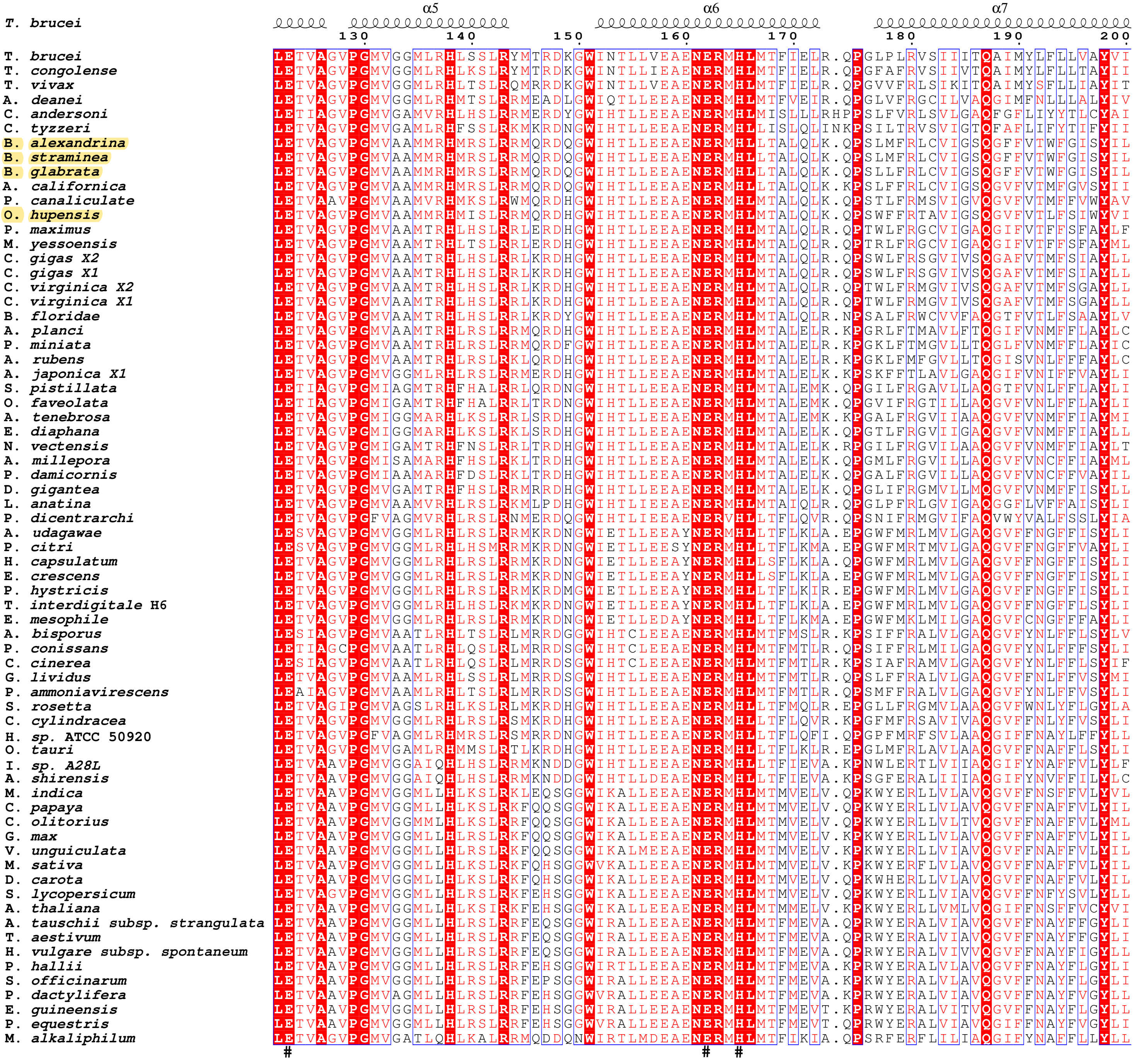

T. brucei
α5
α6
α7
130
140
150
160
170
180
190
200
T. brucei
T. congolense
T. vivax
A. deanei
C. andersoni
C. tyzzeri
B. alexandrina
B. straminea
B. glabrata
A. californica
P. canaliculate
O. hupensis
P. maximus
M. yessoensis
C. gigas X2
C. gigas X1
C. virginica X2
C. virginica X1
B. floridae
A. planci
P. miniata
A. rubens
A. japonica X1
S. pistillata
O. faveolata
A. tenebrosa
E. diaphana
N. vectensis
A. millepora
P. damicornis
D. gigantea
L. anatina
P. dicentrarchi
A. udagawae
P. citri
H. capsulatum
E. crescens
P. hystricis
T. interdigitale H6
E. mesophile
A. bisporus
P. conissans
C. cinerea
G. lividus
P. ammoniavirescens
S. rosetta
C. cylindracea
H. sp. ATCC 50920
O. tauri
I. sp. A28L
A. shirensis
M. indica
C. papaya
C. olitorius
G. max
V. unguiculata
M. sativa
D. carota
S. lycopersicum
A. thaliana
A. tauschii subsp. strangulata
T. aestivum
H. vulgare subsp. spontaneum
P. hallii
S. officinarum
P. dactylifera
E. guineensis
P. equestris
M. alkaliphilum
#
#
#

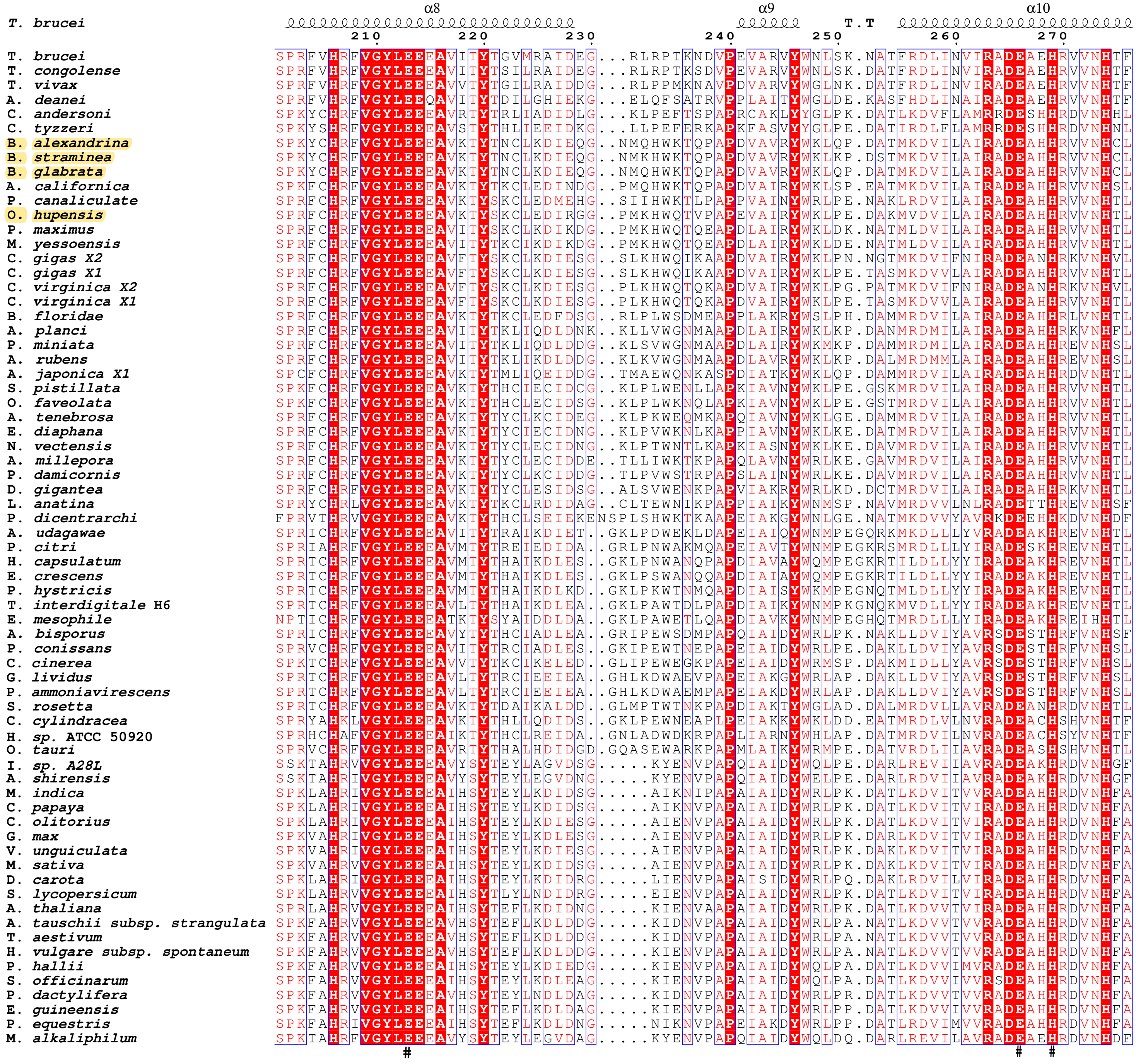

α8
α9
α10
T. brucei
T.T
210
220
230
240
250
260
270
T. brucei
T. congolense
T. vivax
A. deanei
C. andersoni
C. tyzzeri
B. alexandrina
B. straminea
B. glabrata
A. californica
P. canaliculate
O. hupensis
P. maximus
M. yessoensis
C. gigas X2
C. gigas X1
C. virginica X2
C. virginica X1
B. floridae
A. planci
P. miniata
A. rubens
A. japonica X1
S. pistillata
O. faveolata
A. tenebrosa
E. diaphana
N. vectensis
A. millepora
P. damicornis
D. gigantea
L. anatina
P. dicentrarchi
A. udagawae
P. citri
H. capsulatum
E. crescens
P. hystricis
T. interdigitale H6
E. mesophile
A. bisporus
P. conissans
C. cinerea
G. lividus
P. ammoniavirescens
S. rosetta
C. cylindracea
H. sp. ATCC 50920
O. tauri
I. sp. A28L
A. shirensis
M. indica
C. papaya
C. olitorius
G. max
V. unguiculata
M. sativa
D. carota
S. lycopersicum
A. thaliana
A. tauschii subsp. strangulata
T. aestivum
H. vulgare subsp. spontaneum
P. hallii
S. officinarum
P. dactylifera
E. guineensis
P. equestris
M. alkaliphilum
#
#
#

```
T. brucei                          η3
                                   280       290       300       310       320
T. brucei                          ADMHEKRLQNSVNPFVVLKKNPEEMYSNQPSGKTRTDFGSEGAKTASNVNKHV.....
T. congolense                      ADMHEHHLQNSVNPFVVLKKNPAELYNDQGAIKGAAACAAGATTKSS...........
T. vivax                           ADMHAKRLQNCVNPFVVLKTNPELLYANAAPVADKTMSERESGLKDGKLSH.......
A. deanei                          ADMHSHNLCRNPNPFAVLKQTDSK..................................
C. andersoni                       AILKPG.DPNPFASKV..........................................
C. tyzzeri                         ADIRLN.GE.................................................
B. alexandrina                     ASLRKD.DFNPYKPGH..........................................
B. straminea                       ASLRKD.DFNPYKPGQ..........................................
B. glabrata                        ASLRKD.DFNPYKPGH..........................................
A. californica                     ASLKKD.EFNPYKPGK..........................................
P. canaliculate                    ASLKKD.EFNPYKPGH..........................................
O. hupensis                        ASLKAN.QFNPYKPGQ..........................................
P. maximus                         ASLKED.DYNPYKPGK..........................................
M. yessoensis                      ASLKED.DYNPYKPGK..........................................
C. gigas X2                        ASMKTD.QYNPFLPGQ..........................................
C. gigas X1                        ASMKED.EYNPYEPGK..........................................
C. virginica X2                    ASLPTD.KYNPFLPGQ..........................................
C. virginica X1                    ASMKED.EYNPYEPGK..........................................
B. floridae                        SDLDKD.DPNPFQPGK..........................................
A. planci                          SDLKPD.DSNPFPPGQ..........................................
P. miniata                         SDLKPD.ESNPFPPGQ..........................................
A. rubens                          SDLKSD.DSNPFPPGQ..........................................
A. japonica X1                     GTLNLT.DQNPFPPGK..........................................
S. pistillata                      SSLPLC.APNPFHPGEKKL.......................................
O. faveolata                       SSLPLC.APNPFLPGEKKL.......................................
A. tenebrosa                       GTINPH.EPNPFLPGQRKL.......................................
E. diaphana                        SSIHLD.DPNPFHPGQRKL.......................................
N. vectensis                       SSIHLD.DPNPFFPGQRKL.......................................
A. millepora                       SSIHLN.EKNPFSPGQKKL.......................................
P. damicornis                      SSIHLN.EKNPFSPGQKKL.......................................
D. gigantea                        ASMHLD.QQNPFEPWERKL.......................................
L. anatina                         ANNMRN.PHVHNQFKPGDKAINADQCRHTGPCKCKV......................
P. dicentrarchi                    ADDYTQ.QQPNPYSPEQ.........................................
A. udagawae                        GNLQQNADPNPYAAKYKDPSKPHPTKGIENLKPTGWERDEII................
P. citri                           GNLDQKNDPNPYVSEYKKGTKPHPTKDLSYYRPHGWERDDAI................
H. capsulatum                      ANLKQGVDPNPYASKYDNPEAPHPTKSAEIVKPTGWERDEVI................
E. crescens                        ANLNQAVDPNPYAVKYDNPDEPHPTKSAAIIKPTGWERKDVL................
P. hystricis                       ANLNQGTDPNPYASKYDDPKEVRPTKTAEVVKPTGWERDEVL................
T. interdigitale H6                ANLNQKIDPNPYAAKYENPEKPHPTKSAEIVKPTGWDRQDVI................
E. mesophile                       ANLKAE.DPNPFVSVYKDPTKPHPGPSIEHIKSKGWEREDVI................
A. bisporus                        ANLDQRSDVNPFALREPDMHVKGSRVEFKRQESEEYAKVSHEMMEEHLESAKKTNGGL
P. conissans                       ANLNHSTDVNPFALREPDMLVKGQKIEFQRSESEEYVKQSHDLMTKQRVKATDS....
C. cinerea                         ANLNHDTDVNPFALREPDMHTKGKKIEFERHEAEAYIKESHELLGKK...........
G. lividus                         ANLNPAADVNPFALSEPDMFVKGKKLGFERSEAEKYVQESNDSLSRRRAERKH.....
P. ammoniavirescens                ANLNQSTDVNPFAFSEPDMLVKGKKLGFERSEAEKYVQETHELLSRRRAEIKH.....
S. rosetta                         S.TLKTTDVNPYVSGK..........................................
C. cylindracea                     GNMGKE.QTNPFDKHSHTVP......................................
H. sp. ATCC 50920                  SQMKAS.DENPFVPGTHQIS......................................
O. tauri                           SSMGIR.QANPFHVGHTELPENFVDPPPGFVPEHCR......................
I. sp. A28L                        ADELKSGARK................................................
A. shirensis                       ADELKARK..................................................
M. indica                          SDVQVQGKELRDAPAPVGYH......................................
C. papaya                          SDIHYQGKELREAPAPLGYH......................................
C. olitorius                       SDIHFQGKELREAPAPLGYH......................................
G. max                             SDIHFQGKELREAPAPIGYH......................................
V. unguiculata                     SDIHFQGKELREAPAPIGYH......................................
M. sativa                          SDIHFHGKELRDAPAPLGYH......................................
D. carota                          SDIHFEGKELRDAPAPLGYH......................................
S. lycopersicum                    SDIHYQGKKLQEAAAPIGYH......................................
A. thaliana                        SDIRNQGKELREAAAPIGYH......................................
A. tauschii subsp. strangulata     SDVYYQGMQLKATPAPIGYH......................................
T. aestivum                        SDVYYQGMQLKATPAPIGYH......................................
H. vulgare subsp. spontaneum       SDVYYQGMKLKTAPAPIGYH......................................
P. hallii                          SDVHYQGMELKETPAPIEYH......................................
S. officinarum                     SDIHFQGMQLKETPAPLGYH......................................
P. dactylifera                     SDVHYQGMNLKEAPAPLGYH......................................
E. guineensis                      SDVHYQGMNLKQTPAPLGYH......................................
P. equestris                       SDVQYQGMDLKQTPAPVGYH......................................
M. alkaliphilum                    ANQLS.....................................................
```
