# Supplementary material for: The identification of alternative oxidase in intermediate host snails of Schistosoma and its potential role in protecting Oncomelania hupensis against niclosamide-induced stress
Source: Parasit Vectors. 2022 Mar 21;15:97. doi: 10.1186/s13071-022-05227-5 (PMC8935807; doi:10.1186/s13071-022-05227-5)
Supplement: Supplementary file 9 — Additional file 9: Table S4. Mortality rate (%) of snails after WPN treatment, with the AOX inhibitor (SHAM) added at 0 h or 6 h. [file 13071_2022_5227_MOESM9_ESM.docx]

**Additional file 9: Table S4 Mortality rate (%) of snails after WPN treatment when AOX inhibitor (SHAM) was added at 0 h or 6 h**

| **Group** | **6 h** | **12 h** | **24 h** | **48 h** | **χ^2^** | ***P*** |
| --- | --- | --- | --- | --- | --- | --- |
| **H_2_O** | 6.25±6.50 | 3.33±2.37 | 1.25±2.17 | 3.75±4.15 | 1.069 | 0.354 |
| **SHAM** | 6.67±6.24 | 5.00±4.08 | 1.67±2.36 | 12.50±2.50 | 0.751 | 0.415 |
| **Methanol** | 1.67±2.36 | 7.22±6.14 | 2.78±2.08 | 12.50±2.50 | 3.761 | 0.056 |
| **SHAM#** | 6.25±6.50 | 3.33±4.08 | 2.22±2.36 | 10.00±0.00 | 0.858 | 0.491 |
| **Methanol#** | 6.25±6.50 | 2.22±2.36 | 2.22±2.36 | 4.44±5.00 | 0.381 | 0.764 |
| **0.1 WPN** | 5.00±7.07 | 25.00±12.25* | 47.50±13.46*^ab^ | 91.67±6.24*^abc^ | 151.017 | 0.000 |
| **0.1 WPN+SHAM** | 20.00±4.08*^d^ | 50.00±4.08*^ad^ | 71.25±12.93*^ad^ | 100.00±0.00*^abcd^ | 115.669 | 0.000 |
| **0.1 WPN+SHAM#** | 5.00±7.07 | 51.67±4.71*^ad^ | 65.00±4.08*^ad^ | 100.00±0.00*^abcd^ | 44.453 | 0.000 |

# SHAM or methanol in the corresponding group was added at 6 h post-treatment;

* significant difference in the mortality between this group and H_2_O group at the same time point (*P* < 0.05);

a, b, and c indicated the mortality at the time was significantly higher than that at 6 h, 12 h, and 24 h for the same group, respectively (*p* < 0.05);

d indicated significant difference in the mortality at the time between the WPN+SHAM group or WPN+SHAM# group and the WPN group (*p* < 0.05)
